# Evidence for the involvement of ASIC3 in sensory mechanotransduction in proprioceptors

**DOI:** 10.1038/ncomms11460

**Published:** 2016-05-10

**Authors:** Shing-Hong Lin, Yuan-Ren Cheng, Robert W. Banks, Ming-Yuan Min, Guy S. Bewick, Chih-Cheng Chen

**Affiliations:** 1 Institute of Biomedical Sciences, Academia Sinica, 128 Academia Road, Section 2, Taipei 115, Taiwan; 2 Department of Life Science, National Taiwan University, Taipei 106, Taiwan; 3 School of Biological and Biomedical Sciences, University of Durham, Durham DH1 3LE, UK; 4 School of Medicine, Medical Sciences and Nutrition, University of Aberdeen, Aberdeen AB25 2ZD, UK; 5 Taiwan Mouse Clinic-National Comprehensive Mouse Phenotyping and Drug Testing Center, Academia Sinica, Taipei 115, Taiwan

## Abstract

Acid-sensing ion channel 3 (ASIC3) is involved in acid nociception, but its possible role in neurosensory mechanotransduction is disputed. We report here the generation of *Asic3-knockout/eGFPf-knockin* mice and subsequent characterization of heterogeneous expression of ASIC3 in the dorsal root ganglion (DRG). ASIC3 is expressed in parvalbumin (Pv+) proprioceptor axons innervating muscle spindles. We further generate a floxed allele of *Asic3* (*Asic3*^*f/f*^) and probe the role of ASIC3 in mechanotransduction in neurite-bearing Pv+ DRG neurons through localized elastic matrix movements and electrophysiology. Targeted knockout of *Asic3* disrupts spindle afferent sensitivity to dynamic stimuli and impairs mechanotransduction in Pv+ DRG neurons because of substrate deformation-induced neurite stretching, but not to direct neurite indentation. In behavioural tasks, global knockout (*Asic3*^*−/−*^) and *Pv-Cre::Asic3*^*f/f*^ mice produce similar deficits in grid and balance beam walking tasks. We conclude that, at least in mouse, ASIC3 is a molecular determinant contributing to dynamic mechanosensitivity in proprioceptors.

## Abstract (web summary)

Acid-sensing ion channel 3 (ASIC3) is known to play a role in nociception, but its role in low threshold neurosensory mechanotransduction is unclear. Here, the authors target ASIC3 expression in dorsal root ganglion parvalbumin positive neurons and find ASIC3 contributes to dynamic proprioception responses.

## Graphical abstract

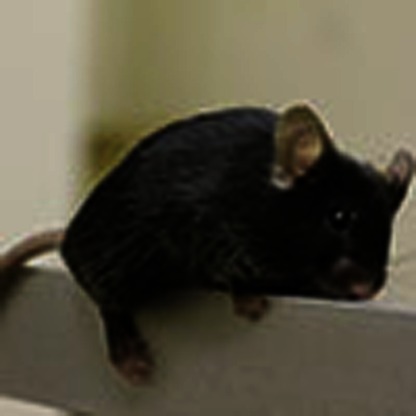

Members of the degenerin/epithelial sodium channel (DEG/ENaC) family have been demonstrated as essential mechanotransducers in the nematode *Caenorhabditis elegans*^1^ and fly *Drosophila melanogaster*^2^. As a mammalian homologue, the amiloride-sensitive acid-sensing ion channel 3 (ASIC3) may thus play a role in mechanosensation^3^,^4^,^5^. Results of conventional knockout studies have shown that mice totally lacking *Asic3* (*Asic3*^*−/−*^) have deficits in neurosensory mechanotransduction of tissues such as skin, stomach, colon, venoatrial junction and cochlea^6^,^7^,^8^,^9^. Controversy exists, however, as enhanced mechanotransduction was found in *Asic3*^*−/−*^ mice in both *in vitro* single-fibre recording and *in vivo* mechanical nociception assays^6^,^8^,^10^,^11^. Moreover, *Asic3* knockout had no effect on currents activated mechanically by direct soma contact in dissociated dorsal root ganglion (DRG) neurons^12^. This variability in results from different paradigms might reflect the fact that ASIC3 is expressed in a heterogeneous population of DRG neurons^13^,^14^. Electrophysiological recording combined with retrograde labelling and immunofluorescence in knockout studies have elucidated the channel kinetics and acid-sensing function of ASIC3 in skin^15^, muscle^16^ and cardiac^17^ afferents. As ASIC channels are trimeric, it is possible that different populations of DRG sensory neurons might express different compositions of homo- and/or heterotrimeric channels containing ASIC3, according to the different sensory modalities of the neurons and their projections to different peripheral organs^4^,^18^. Thus, to elucidate the role of ASIC3-containing channels in mechanotransduction is proving challenging. One possible approach would be to examine the role of ASIC3 in specific mechanoreceptors that carry out well-defined mechano-sensing properties. However, concerns about antibody specificity and the heterogeneous nature of ASIC3-containing channels can restrict our ability to determine the role of ASIC3 in mechanotransduction. For instance, ASIC3 immunoreactivity has been detected in many types of low-threshold mechanoreceptors, but electrophysiological approaches combined with retrograde tracing have so far failed to identify ASIC3 expression in proprioceptors (low-threshold mechanoreceptors responsible for proprioception) or other mechanoreceptors^6^,^9^,^19^. We report here a genetic axonal tracing approach to probe ASIC3 function in a small and homogeneous population of DRG Pv+ neurons. Conditional knockout of *Asic3* in these neurons not only greatly attenuates acid sensing but also eliminates a specific mechano-sensing response in isolated neurons. The physiological importance of ASIC3 in these Pv+ neurons is further supported by deficits in proprioception in *ex vivo* muscle-nerve recording and *in vivo* locomotion behaviour tests on conditional *Asic3* knockout mice.

## Results

### Heterogeneous expression of ASIC3 in the mouse DRG

**Figure 1:**
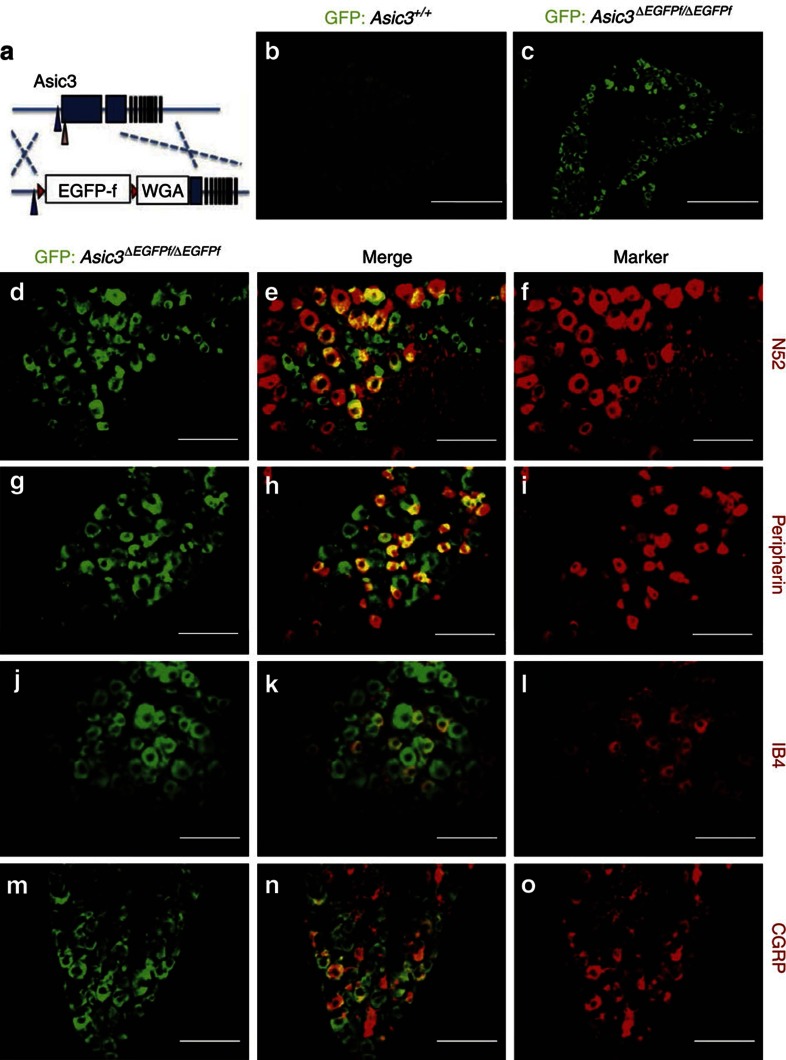
Immunofluorescence staining of GFP in ASIC3-expressing DRG neurons in *Asic3-KO/eGFP-f-KI* (*Asic3*Δ^*EGFPf/EGFPf*^) mice. (**a**) Illustration of the *EGFPf*^*floxed*^*-WGA* construct targeted in *Accn3* allele in mice. Blue and pink arrowheads indicate the position of the transcription and translation start sites of mouse *Accn3*, respectively. Red triangles represent the loxP sites. Blue boxes indicate the exons. (**b**) GFP signal was very low in wild-type lumbar DRG (scale bar, 50 μm). (**c**) Expression of GFP was much higher in the *Asic3*Δ^*EGFPf/EGFPf*^ lumbar DRG (scale bar, 50 μm). (**d**–**o**) Co-localization of GFP signals with: (**d**–**f**) myelinated neuron marker N52, (**g**–**i**) small nociceptor marker peripherin, (**j**–**l**) non-peptidergic nociceptor marker IB4 and (**m**–**o**) peptidergic nociceptor marker CGRP in the *Asic3*Δ^*EGFPf/EGFPf*^ lumbar DRG are illustrated (scale bar, 100 μm).

To map the expression of ASIC3 in DRG neurons and their corresponding peripheral targets, we first knocked in a membrane-tagged farnesylated enhanced green fluorescent protein (eGFP-f) into the mouse *Accn3* locus (Supplementary Fig. 1). In these *eGFP-f* knock-in (*Asic3*Δ^*EGFPf/EGFPf*^) mice (Fig. 1a), immunostaining for eGFP indicated that ASIC3 is expressed in 29.6% (589/1,987) of DRG neurons (Fig. 1b,c). The knock-in membrane-tagged eGFP signals in DRG are further confirmed by confocal microscopy (see *z*-axis stack results in Supplementary Fig. 1f–h). Consistent with previous reports that ASIC3 is expressed in both large- and small-diameter DRG neurons^6^, we found 48.7% (645/1,345) of large-diameter (N52-positive) myelinated neurons (Fig. 1d–f) and 39.2% (897/2,286) of small-diameter (peripherin-positive) neurons (Fig. 1g–i) showed expression of GFP. Both peptidergic and non-peptidergic nociceptors expressed ASIC3, as 22.9% (240/1,024) of IB4-positive (Fig. 1j–l) and 26.8% (420/1,410) of CGRP-positive neurons (Fig. 1m–o) showed expression of eGFP.

### ASIC3 is expressed in most parvalbumin-positive DRG neurons

**Figure 2:**
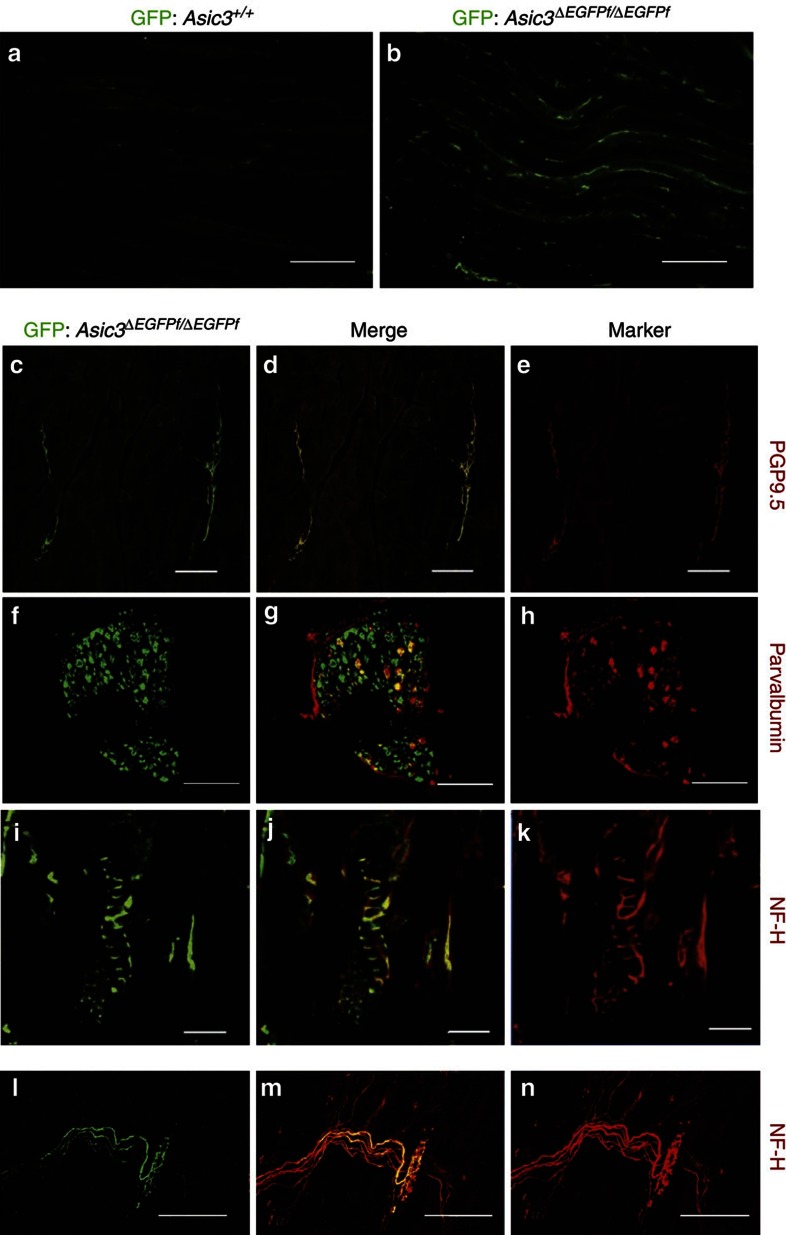
ASIC3-expressing DRG afferent neurons in muscle and proprioceptors. (**a**,**b**) eGFP immunofluorescence in horizontal sections of gastrocnemius muscle is low in wild-type but high in *Asic3*Δ^*EGFPf/EGFPf*^ mice. Scale bar, 200 μm. (**c**–**e**) eGFP and the nerve marker PGP9.5 co-localize in the nerves of gastrocnemius muscle (transverse section: scale bar, 100 μm). (**f**–**h**) eGFP is present in most (∼90%) parvalbumin positive, large diameter neurons in the lumbar DRG of the *Asic3*Δ^*EGFPf/EGFPf*^ mice, as well as many smaller, parvalbumin-negative somata. Scale bar, 200 μm. (**i**–**k**) Co-localization of GFP and myelinated-nerve marker neurofilament heavy chain (NF-H) in annulospiral endings characteristic of spindles in the soleus muscle of an *Asic3*Δ^*EGFPf/EGFPf*^ mouse (scale bar, 50 μm). (**l**–**n**) Co-localization of GFP and NF-H in the Golgi tendon organs from an *Asic3*Δ^*EGFPf/EGFPf*^ mouse embryo's gluteus muscle (scale bar, 50 μm).

ASIC3 shows only a restricted expression in certain kinds of cutaneous afferents, but is much more widely expressed in muscle afferents including low- and high-threshold mechanoreceptors, metaboreceptors and nociceptors^20^,^21^,^22^,^23^. We therefore examined the expression of eGFP in the gastrocnemius muscle in *Asic3*Δ^*EGFPf/EGFPf*^ mice. We examined the extent of co-localization of eGFP expression with the pan-neuronal marker PGP9.5 and found eGFP signals, as expected, arose from nerve bundles in the gastrocnemius muscle (Fig. 2a–e). Parvalbumin is a well-known marker for low-threshold mechanoreceptors, including proprioceptors such as muscle-spindle afferents^24^, but previous studies in rats to test for co-localization between ASIC3 and parvalbumin have been inconclusive^20^,^25^. We, therefore, examined the extent of co-localization of ASIC3 and parvalbumin genetically in lumbar DRG of *Asic3*Δ^*EGFPf/EGFPf*^ mice, where eGFP expression is driven by the ASIC3 promoter. Strikingly, immunostaining for parvalbumin in *Asic3*Δ^*EGFPf/EGFPf*^ DRG neurons indicated that the great majority (∼89.5%) of Pv+ DRG neurons showed eGFP expression (517/583 from *N*=3 mice, independent trials; Fig. 2f–h). In addition, eGFP signals co-localized closely with the myelinated nerve marker, neurofilament heavy chain (NF-H) in both the muscle spindles of soleus muscle and the Golgi tendon organs (GTO) of gluteus muscle (Fig. 2i–n). The existence of ASIC3-containing nerve fibres in the gastrocnemius muscle is consistent with previous reports^23^, suggesting this ion channel is involved in the development of acid-induced chronic mechanical hyperalgesia and/or the sensing of muscle metabolites and ischaemia. In contrast, the existence of ASIC3 expression in muscle-spindle afferents is intriguing, because they are large-diameter, low-threshold mechanoreceptors that are thought to exclusively detect the changes in the length and velocity of muscle stretch. We thus hypothesized that ASIC3, as a mammalian homologue of *mec-4* in *C. elegans*^3^, may play a role in sensing muscle/mechanical stretch in these Pv+ DRG proprioceptors.

### Conditional knockout of ASIC3 in DRG Pv+ neurons

**Figure 3:**
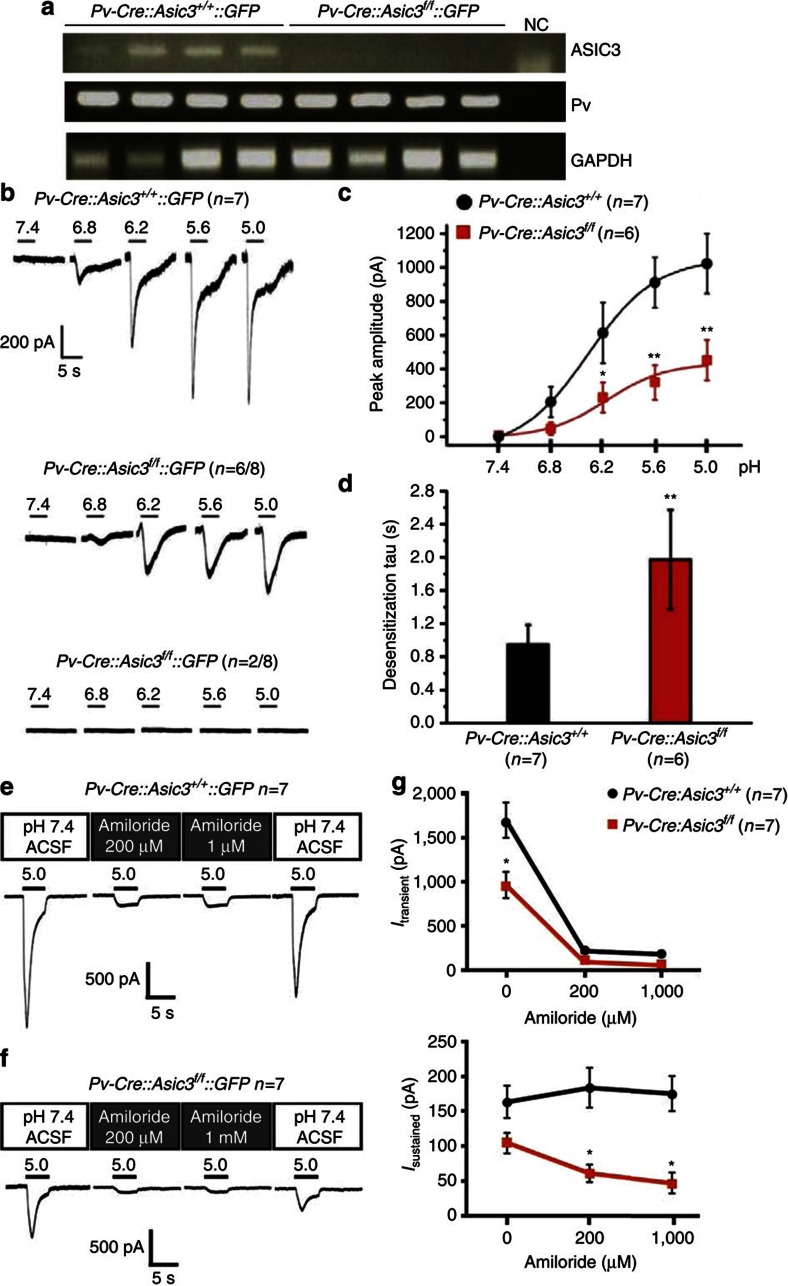
Conditional knockout of *Asic3* in DRG Pv+ neurons. (**a**) Single-cell RT–PCR indicated that every GFP-positive DRG neuron showed expression of parvalbumin (Pv) and ASIC3 transcripts in *Pv-Cre::Asic3*^*+/+*^*::GFP-reporter* mice, whereas ASIC3 transcripts were eliminated in the *Pv-Cre::Asic3*^*f/f*^*::GFP-reporter* samples, showing the *Asic3* gene expression was successfully disrupted. (**b**) Acid-induced inward currents (*I*_acid_) were impaired (*n*=6/8) or completely undetectable (*n*=2/8) in *Asic3*-null Pv+ DRG neurons. (**c**) Even in responsive neurons, the peak amplitudes of acid-induced currents were significantly smaller (by ∼50%) in *Asic3*-null Pv+ DRG neurons than in wild-type controls at all pHs below 6.8 (pH 6.8, *U*=9.0, *P*=0.053; pH 6.2, *U*=30.0, *P*<0.05; pH 5.6, *U*=35.0, *P*<0.01; and pH 5.0, *U*=2.0, *P*<0.01). (**d**) In these same neurons, at pH=5.0, the desensitization time constant of *I*_acid_ in proprioceptors was also substantially longer in *Asic3* knockouts (Mann–Whitney *U*-test, *U*=3.0, *P*<0.01). (**e**) Only the transient phase of the acid (pH 5.0)-induced current was inhibited by amiloride (200 μM or 1 mM) in wild-type Pv+ DRG neurons. However, (**f**) both transient and sustained phases of the acid (pH 5.0)-induced current were inhibited by the same doses of amiloride in *Asic3*-null neurons. These data are summarized in **g**. For transient current, two-way ANOVA with repeated measurement indicated significant effect of genotype (F_(1,12)_=10.764, *P*<0.01), dose of amiloride (F_(2,24)_=82.483, *P*<0.01) and their interaction (F_(2,24)_=5.404, *P*<0.05). *Post hoc* comparison indicated significant difference between genotypes only in amiloride dose 0 μM (*t*=4.616, *P*<0.05). For sustained current, two-way ANOVA with repeated measurement indicated significant effect of genotype (F_(1,12)_=14.252, *P*<0.01) and interaction (F_(2,24)_=3.801, *P*<0.05) but not dose of amiloride (F_(2,24)_=1.283, *P*=0.295). *Post hoc* comparison of interaction indicated significant difference between genotypes in amiloride dose 200 μM (*t*=3.877, *P*<0.05) and 1,000 μM (*t*=4.024, *P*<0.05). Data are mean±s.e.m. *P<0.05, **P<0.01 between groups.

To achieve cell-type-specific elimination of ASIC3, we next generated another mouse line with a floxed allele of mouse *Accn3* (*Asic3*^*f/f*^) to enable a Cre-mediated conditional knockout (Supplementary Fig. 2). As a comparative test of successful specific knockout, we used *Nav1.8-Cre*^26^ and *Parvalbumin-Cre* (*Pv-Cre*) mice (Jackson Laboratory, Stock Number: 008069) to selectively target a knockout of *Asic3* in DRG nociceptors and low-threshold mechanoreceptors, respectively. We first examined the Cre-mediated recombination by crossing the *Cre*-mice with *CAG-STOP*^*floxed*^*-GFP* (*GFP-reporter*) mice. In *Pv-Cre::GFP-reporter* mice, GFP signals co-localized well (∼87%) with parvalbumin-expressing DRG neurons (Supplementary Fig. 3a–c). In *Nav1.8-Cre::GFP-reporter* mice, the GFP signals did not overlap with parvalbumin-expressing DRG neurons, indicating that parvalbumin and Nav1.8 are expressed by two different neuron populations in DRG (Supplementary Fig. 3d–f). In the two Cre-mediated *Asic3* conditional knockout lines (*Nav1.8-Cre::Asic3*^*f/f*^ and *Pv-Cre::Asic3*^*f/f*^), PCR analyses on genomic DNA suggested that the floxed *Accn3* is faithfully excised in a Cre-dependent manner (Supplementary Fig. 3g,h). By crossing the *Pv-Cre::Asic3*^*f/f*^ lines with *GFP-reporter* mice, we were able to visualize Cre-expressing DRG neurons in the culture by fluorescence microscopy. This would be expected to allow us to target Pv+ neurons with eGFP expression, so as to study cell-type-specific ASIC3 channel electrophysiological properties. To test the validity of this approach, single cells with an eGFP signal were harvested and subjected to reversed transcription–PCR (RT–PCR) analysis using intron-spanning, nested primers targeting mouse ASIC3 and parvalbumin. Cultures from *Pv-Cre::Asic3*^*+/+*^*::GFP-reporter* mice showed that every GFP-positive neuron (*N*=4/4) expressed both parvalbumin and ASIC3, whereas, conversely, ASIC3 transcripts were not found in DRG neuron samples from homozygous floxed (*Pv-Cre::Asic3*^*f/f*^*::GFP*)-knockout mice (Fig. 3a). To test whether ASIC3 in these cultured DRG Pv+ neurons form functional ion channels and are sensitive to reductions in extracellular pH, we conducted whole-cell patch-clamp recording to measure the acid-evoked inward current. In voltage clamp mode, extracellular acid stimulation induced robust inward currents in wild-type GFP-positive Pv+ neurons in a pH-dependent manner, whereas in the GFP-positive ASIC3-null neurons, the acid-induced currents were either totally abolished (2/8) or significantly decreased (6/8; Fig. 3b). For these six acid-sensitive *Asic3* knockout Pv+ neurons, the acid-induced peak current amplitudes were significantly lower at all sampled values of pH below 6.8 (*P*<0.01) as compared with those in wild-type Pv+ neurons (Fig. 3c). At pH=5.0, acid-induced current desensitization was significantly slower in *Asic3*-null Pv+ neurons as compared with wild-type Pv+ neurons (*P*<0.01, Fig. 3d). We next investigated the basis of the acid-induced inward current (which was also ASIC-like) that persisted in most *Asic3*-null Pv+ DRG neurons. We screened for the expression of other ASIC subtypes and Piezo1/2 in Pv+ DRG neurons using single-cell RT–PCR. Piezo1/2 were included due to the current interest in Piezo1/2 as mechanosensitive ion channels in DRGs^27^,^28^,^29^,^30^. Results indicated that ASIC1b, ASIC2b and Piezo2 but not ASIC1a, ASIC2a or Piezo1 were also expressed in Pv+ DRG neurons (Supplementary Fig. 4). Moreover, amiloride (200 μM) dramatically inhibited the transient phase of the acid-induced inward current evoked at pH 5.0 in both wild-type and *Asic3*-null Pv+ neurons (Fig. 3e–g). Overall, these results indicate that ASIC3 plays a major role in acid-sensation in DRG Pv+ neurons. In addition, it raises the possibility it may function as a homotrimer or, as a heterotrimer with ASIC1b and/or ASIC2b.

### A mode-specific force-sensing role for ASIC3 in DRG neurons

**Figure 4:**
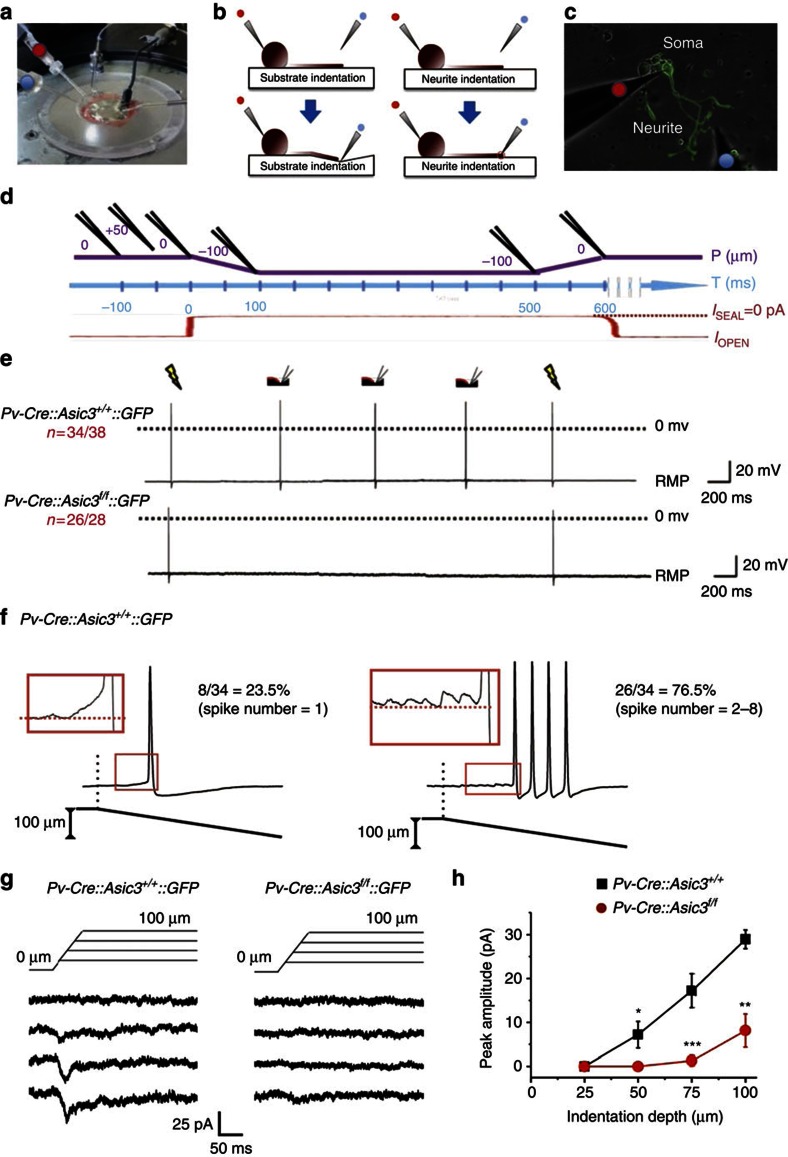
ASIC3 mediates substrate deformation-driven neurite stretch (SDNS)-induced firing in Pv+ DRG neurons. (**a**) Experimental setup for whole-cell patch-clamp recording. The red dot indicates the recording pipette and the blue dot indicates the indentation pipette. (**b**) Schematic of stretching (left panels: substrate indentation, SDNS) or pressing on (right panels: neurite indentation, DNI) a neurite grown on an elastic substrate. (**c**) Cultured on a PDMS substrate, the soma of a suitable GFP-positive Pv+ DRG neuron was patched with a recording pipette (red dot). An indentation pipette (blue dot) was positioned 300 μm away from the patched soma and on top of (DNI) or 15–25 μm away from (SDNS) the neurite. (**d**) Experimental protocol for mechanical stretching. The temporospatial relationship of the indentation pipette (in black above the purple line), the surface of the PDMS substrate (purple line) are illustrated and aligned with the recording time (blue line). The leak current of the indentation pipette (red line) monitors when the pipette contacts the PDMS surface. The leak current when sealed (*I*_sealed_) is taken as 0 pA (see the Methods for details). (**e**) Each SDNS generated an action potential in wild type, but not *Asic3*-null Pv+ DRG neurons. RMP, resting membrane potential. Electrical stimulation via the patch electrode (yellow flashes) also generates a similar action potential response. (**f**) In 34/38 wild-type Pv+ DRG neurons, SDNS induced single (*n*=8) or a train of (*n*=26) action potentials. (**g**) SDNS-induced mechanosensitive currents were recorded via voltage clamp. In wild-type Pv+ DRG neurons, indentation depths of 25, 50, 75 and 100 μm generated progressively increasing inward currents. The current was dramatically decreased in *Asic3*-knockout Pv+ DRG neurons. (**h**) Comparison of the SDNS-induced mechanical currents in wild-type and *Asic3* KO Pv+ DRG neurons. Significant differences with genotype was (Mann–Whitney *U*-test) are found at indentation depths of 50, 75 and 100 μm (*U*=30.0, *P*<0.05; *U*=10.0, *P*<0.01; *U*=14.5, *P*<0.01, respectively). Data are mean±s.e.m. *P<0.05, **P<0.01, ***P<0.001 between groups.

Compared with Nav1.8, which is expressed in 49.2% of DRG neurons^31^, parvalbumin is expressed in a much smaller proportion (5% of total DRG neurons and only 14.9% of the large, myelinated neurons, see Supplementary Fig. 3i–k) of the DRG neuron population. These Pv+ DRG neurons include proprioceptors whose role is to sense the stretch and mechanical force of the muscle through spindles or GTO, respectively, and transmit the information to motor neurons and premotor interneurons in the spinal cord^32^ (Supplementary Fig. 3l–n). We next tested whether ASIC3 could contribute to mechanotransduction following neurite stretch in eGFP-positive/Pv+ neurons. Neurite-bearing DRG Pv+ neurons were grown on fibronectin-coated elastic substrate, polydimethylsiloxane (PDMS), and neurite stretch was generated by substrate indentation with a glass pipette 15–25 μm away from the GFP-positive neurite via an electronically controlled micromanipulator (Fig. 4a-d). The elastic substrate allowed us to stretch single neurites without direct contact with the glass probe^33^. Substrate deformation-driven neurite stretch (SDNS) generates a different modality of mechanical force from the direct probe indentation used in most previous studies. In particular, with SDNS, the mechanical force is conducted to the neurite by extracellular matrix (ECM) tethering (see Supplementary Movies 1 for details), which is required to transduce mechanical stimuli in low-threshold mechanoreceptors^34^. In 38 wild-type Pv+ neurons recorded, 34 responded consistently (∼90% success rate) with stretch-induced action potentials (APs) across three successive substrate-indentation trials (Fig. 4e). The SDNS-induced responses for each stretch were either a single AP (8/34) or trains of APs (2–8 spikes, 26/34; Fig. 4f). In contrast, in most *Asic3*-null Pv+ neurons (26/28), neurite stretches failed to generate APs in any of the three trials. In the remaining 2 (<8%), the neurons produced a single AP, but only on the first stretch (Supplementary Fig. 5). A *χ*^2^ contingency test indicated there was a significant difference in the AP-firing rate between wild-type and cKO mice (*χ*2=44.07, *P*<0.01). In the four wild-type Pv+ neuron ‘non-responders', three produced an AP on the first-trial only, whereas one failed to produce any APs, across all three neurite stretch trials (see Supplementary Fig. 5a,b for details). All non-responsive neurons were otherwise healthy. Control trials of electric stimulation were performed before and after the three SDNS trials to ensure that all the neurons had the capacity to generate APs. There was no difference in AP parameters (threshold, overshoot amplitude, after-hyperpolarization amplitude and duration) between electrically and SDNS-driven APs. ASIC3 does not appear to contribute directly to neuronal-membrane excitability or AP profile, as there was no difference between wild-type and ASIC3-null Pv+ neurons in measured parameters either for directly electrically induced APs or in rheobase analysis (Supplementary Fig. 6). However, marked differences emerged when we probed the force-sensing role of ASIC3 in the SDNS trials. When we compared SDNS-induced mechanosensitive inward current between genotypes in voltage clamp, inward current was dramatically decreased in *Asic3*-null Pv+ DRG neurons (Fig. 4g). Significant genotype differences in SDNS-induced current peak amplitude were immediately apparent at the minimum indentation depth to elicit measurable current, 50 μm (Fig. 4h).

**Figure 5:**
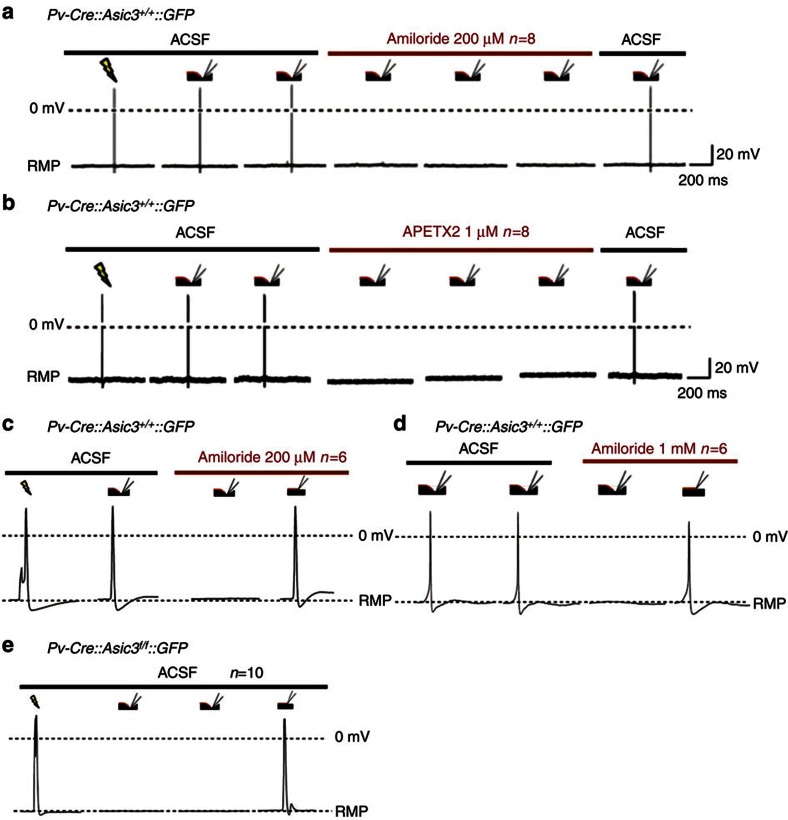
Pharmacological blockade of ASIC3 eliminated SDNS-induced action potentials in wild-type Pv+ DRG neurons. (**a**) Amiloride (200 μM), a broad-spectrum ASIC/ENaC channel blocker, blocked SDNS-induced action potentials in wild-type Pv+ DRG neurons. (**b**) APETx2 (1 μM), a potent and selective inhibitor of ASIC3, also blocked SDNS-induced action potentials in wild-type Pv+ DRG neurons. (**c**,**d**) In contrast, DNI still evoked action potentials in the presence of amiloride (200 μM or 1 mM) in wild-type DRG proprioceptors. (**e**) Similarly, DNI also generated action potentials in *Asic3* knockout Pv+ DRG neurons. RMP, resting membrane potential.

As described in the previous experiment, SDNS consistently generates APs in wild-type Pv+ DRG neurons. However, this response was blocked in the presence of either the broad-spectrum DEG/ENaC inhibitor amiloride (200 μM; Fig. 5a) or the ASIC3-specific blocker APETx2 (1 mM; Fig. 5b). In our PDMS substrate culture system, another mode of mechanical stimulation is possible: that of direct neurite indentation (DNI). Thus, for comparison, we then asked if these antagonists blocked DNI-evoked responses in the same neurite, with the same flame-polished probe. In wild-type Pv+ neurons, even when SDNS-induced APs were inhibited by amiloride, DNI still induced APs (Fig. 5c,d). In addition, we found DNI also consistently induced APs in all ASIC3-null Pv+ neurons (Fig. 5e). These results indicate ASIC3 is an important determinant of low-threshold mechanosensitive responses in SDNS-activated Pv+ neurons, whereas other mechanosensitive channels are responsible for DNI-induced APs. Thus, they highlight a modality-specific role of ASIC3 in performing mechanotransduction in DRG Pv+ neurons.

### ASIC3 KO impairs dynamic responses in muscle spindles

**Figure 6:**
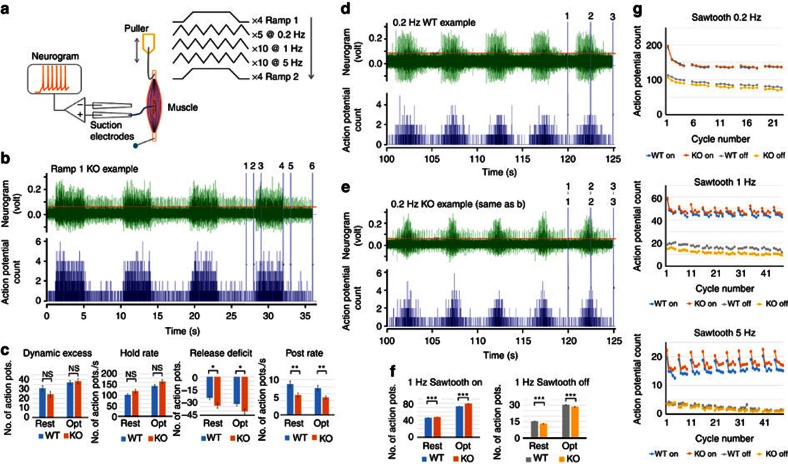
*Asic3* KO enhances muscle proprioceptor dynamic sensitivity. (**a**) Recording arrangement and stretch protocol. The *ex vivo* mouse soleus muscle was pinned to the PDMS at one end and stretched by an electromagnetic puller at the other end. The nerve was drawn into a suction electrode record stretch-evoked activity. A series of stretches was applied at two initial lengths— ‘resting', then ‘optimal' (see the Methods for details). (**b**) Typical responses to ramp stretches in a conditional knockout (KO, *Pv-Cre::Asic3*^*f/f*^) mouse (green trace). Action potentials exceeding the cursor position just above background noise are counted (blue trace below, bin width 0.01 s). The numbered vertical cursors show the phases of the ramp where action potential total counts were determined: 1–2, pre-stretch; 2–3, ramp-on; 3–4, hold; 4–5, ramp-off; 5–6, post-stretch. (**c**) Comparisons at different phases of the ramp of the proprioceptor responses in WT (*Asic3*^*f/f*^) and KO (*Pv-Cre::Asic3*^*f/f*^) mice. Dynamic excess: action potential count during ramp-on exceeding that expected from linear interpolation between pre-stretch and hold rate. Release deficit: action potential count during ramp-off below the linear interpolation between hold and post-stretch rates. (**d**) Typical response to repeated triangular (‘sawtooth') stretch-and-release at 0.2 Hz in a WT mouse (green trace). Numbered vertical cursors mark the stretch (ON/on in **f**,**g**) and release (OFF/off in **f**,**g**) of a group of 5 triangles. (**e**) As in **d**, for the KO (*Pv-Cre::Asic3*^*f/f*^) mouse shown in **b**. (**f**) ON and OFF proprioceptor responses from WT (*Asic3*^*f/f*^) and KO (*Pv-Cre::Asic3*^*f/f*^) mice to 1 Hz sawtooth stretch. (**g**) Mean on and off phase action-potential counts for sawtooth stretches at 0.2, 1 and 5 Hz in WT and KO mouse soleus muscles from resting lengths, showing the consistent differences. The intervals between successive groups of 5 triangular stretches allowed some recovery in firing rate in an otherwise steady adaptation over time. In **c** and **f**: rest, muscles initially at resting length; opt, muscles initially at optimal length. Data are mean±s.e.m. Significance of differences: *P<0.05; **P<0.01; ***P<0.001.

Amiloride-sensitive channels are a major contributor to mechanotransduction in muscle spindles^35^. We therefore examined the impact of *Asic3* deletion on stretch-evoked responses of mouse soleus muscle proprioceptors (Fig. 6). The proximal tendon of excised muscles was fixed in the recording chamber, and stretches applied to the distal tendon. A suction electrode recorded stretch-evoked whole-nerve firing from two initial lengths: at ‘rest'—the *in situ* length when the ankle angle was 90° of dorsi-flexion—and then at ‘optimal' length, where any stretch immediately evoked robust firing but shortening had no effect.

Perhaps surprisingly, given the preceding SDNS results, stretch produced vigorous firing in *Asic3*^*−/−*^ and wild-type muscles (Fig. 6b,d,e). However, *Asic3* deletion significantly affected dynamic responses. For ramps, AP counts were made in five regions—pre-stretch, ramp on, hold, ramp off and post-stretch. The on and off responses were expressed as the difference in counts relative to the linear interpolation between pre-stretch and hold, or hold and post-stretch, phases respectively producing a dynamic excess (on) and release deficit (off). *Asic3*^*−/−*^ mice had a significantly greater release deficit (two-way analysis of variance (ANOVA) with replicates: wild type versus *Asic3*^*−/−*^; from rest: F=5.33, for 1 d.f., *P*<0.03; from optimal: F=4.61, for 1 d.f., *P*<0.04), which persisted into a reduced post-stretch firing rate at both rest and optimal initial lengths (F=8.12, for 1 d.f., *P*<0.01; and F=8.36, for 1 d.f., *P*<0.01, respectively). For triangular stretches, simple AP counts were made for the ‘on' and ‘off' phases. *Asic3*^*−/−*^ muscles had reduced ‘off' responses at 0.2 Hz, which were more evident at 1 Hz. Conversely, they had greater ‘on' responses at 1 and 5 Hz (two-way ANOVA with replicates: wild type versus *Asic3*^*−/−*^; for results of statistical analysis, see Table 1). Thus, the predominant effect of *Asic3* deletion was to enhance stretch-evoked dynamic firing and reduce firing during the release phase, with more pronounced ‘on' responses at higher stretch velocities, but the opposite trend during the release phase. Given the quite striking effects in isolated neurons and more subtle effects *ex vivo*, it was important to determine the overall effects on locomotion and motor co-ordination.

### ASIC3 KO impairs grid- and balance-beam walking

**Figure 7:**
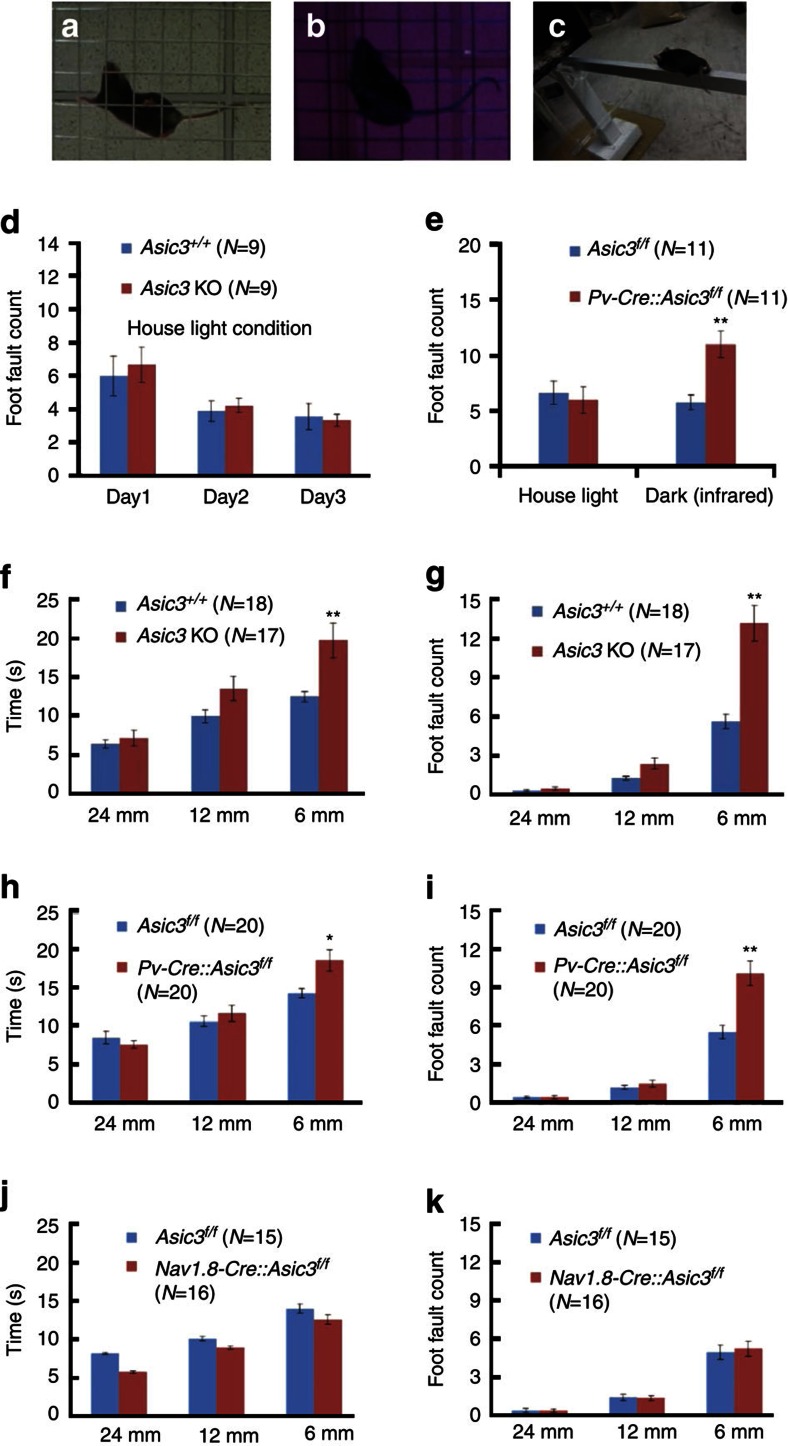
Mice lacking *Asic3* had behavioural deficits in proprioception. (**a**) Grid-walking task in normal light. (**b**) Grid-walking task in the dark, imaged with an infrared-aided camera. (**c**) Balance beam walking task. (**d**) Foot fault errors during 5 min walking on the grid were counted. *Asic3*^*−/−*^ mice showed no difference from *Asic3*^*+/+*^ mice in foot fault counts or learning during three consecutive days of training. Two-way ANOVA with repeated measures: 3 days of training, F _(2,32)_=5.941, *P*<0.01), genotype (F _(1,32)_=0.169, *P*=0.686), interaction (F _(2,32)_=0.129, *P*=0.879), (**e**) Grid walking task foot faults in normal light or in the dark (imaged with an infrared-aided camera). *Pv-Cre::Asic3*^*f/f*^ mice made more errors in the dark (two-way ANOVA, genotype × darkness interaction (F_(1,34)_=4.527, *P*<0.05)). (**f**,**g**) *Asic3*^*−/−*^ mice took longer and also had an elevated error rate in the narrowest beam-walking task under normal lighting. Time: two-way ANOVA with repeated measures for genotype (F_(1,66)_=7.455, *P*<0.05), beam width (F_(2,66)_=43.89, *P*<0.01) and interaction (F_(2,66)_=5.265, *P*<0.01), *post hoc* Holm-Sidak comparison: genotype difference in 6-mm-width trial (*t*=3.962, *P*<0.01); Foot fault: two-way ANOVA with repeated measures for genotype (F_(1,66)_=23.294, *P*<0.01), beam width (F_(2,66)_=140.651, *P*<0.01) and interaction (F_(2,66)_=24.312, *P*<0.01), *post hoc* Holm-Sidak comparison: genotype difference only in the 6-mm-width trial (*t*=8.366, *P*<0.01). (**h**,**i**) A similar deficit occurred in *Pv-Cre::Asic3*^*f/f*^ mice. For the beam traverse time, two-way ANOVA with repeated measures indicated a significant difference with respect to beam width (F_(2,76)_=66.813, *P*<0.01), interaction (F_(2,76)_=6.512, *P*<0.01) but not genotype (F_(1,76)_=2.2194, *P*=0.145). Overall, a *post hoc* Holm-Sidak comparison indicated significant genotype difference in the 6-mm width trial (*t*=3.31, *P*<0.01). For the foot fault count, two-way ANOVA with repeated measures indicated significant difference in the genotype (F_(1,76)_=14.681, *P*<0.01), beam width (F_(2,76)_=159.638, *P*<0.01) and interaction (F_(2,76)_=16.306, *P*<0.01). Again, *post-hoc* Holm-Sidak comparisons indicated significant genotype difference only in the 6-mm-width trial (*t*=6.842, *P*<0.01). (**j**,**k**) *Nav1.8-Cre::Asic3*^*f/f*^ conditional knockout mice did not show behavioural deficits in the balance beam walking task. Two-way ANOVA indicated no effect of genotype (time: F_(1,58)_=2.232, *P*=0.146; foot fault: F_(1,58)_=0.044, *P*=0.836) or interaction (time: F _(2,58)_=0.524, *P*=0.595; foot fault: F _(2,58)_=0.113, *P*=0.893). Data are mean±s.e.m. *P<0.05, **P<0.01.

If ASIC3 is involved in some aspect of proprioception, walking behaviour might be perturbed by these effects of *Asic3* deletion. We first screened behavioural phenotypes in conventional *Asic3*^*−/−*^ mice in two well-known locomotion tasks related to rodent proprioception: the grid-walking and balance beam walking (Fig. 7a–c). Previous studies in our laboratory have demonstrated that these *Asic3*^*−/−*^ mice show normal locomotion in open field and rotarod tasks^21^. In the present study, conventional *Asic3*^*−/−*^ mice again performed well in the grid-walking task. The foot fault count of wild type and knockouts both decreased significantly during three consecutive days of training, with no significant effect of genotype or interaction (Fig. 7d). We speculated whether there might actually be latent and subtle deficiencies in grid walking performance in the *Asic3*^*−/−*^ mice, but other sensory modalities were being used to compensate, particularly visual cues. We, therefore, eliminated visual cues by testing in a darkened room and counting the number of foot faults with the help of an infrared-aided camera. When visual cues were impaired in this way, the foot fault incidence increased in *Pv-Cre::Asic3*^*f/f*^ mice (Fig. 7e). The most sensitive task for detecting sensorimotor deficits in hindlimb function is the beam-walking task^36^. In full-light conditions, three different beam widths, of 24, 12 and 6 mm progressively increased the level of difficulty in the task. For the 6-mm wide beam, *Asic3*^*−/−*^ mice showed increases in both the time (Fig. 7f) and the foot fault count (Fig. 7g) compared with their wild-type siblings when traversing the 1-m-long beam. Consistent with our hypothesis that ASIC3 in proprioceptors plays a role in sensing muscle stretch, *Pv-Cre::Asic3*^*f/f*^ mice showed the same phenotype but not *Nav1.8-Cre::Asic3*^*f/f*^ (Fig. 7h-k). Further, the strategy for *Asic3*^*−/−*^ and *Pv-Cre::Asic3*^*f/f*^ mice to complete the 6-mm beam walking was very different from most of their wild-type siblings. Crawling was observed in 72.5% of *Asic3*^*−/−*^ (versus 18.5% in WT (*Asic3*^*+/+*^) siblings) and 65% in *Pv-Cre::Asic3*^*f/f*^ (versus 15% in WT (*Asic3*^*f/f*^) siblings; see Supplementary Movies 2 and 3). These behavioural deficits in the proprioception tasks in *Asic3*-null mice seem not be explained by direct effects of *Asic3* deletion in the cerebellum or precerebellar nuclei, because immunostaining of GFP in *Pv-Cre::Asic3*^*+/+*^*::GFP-reporter* and *Asic3*Δ^*EGFPf/EGFPf*^ mice indicated that ASIC3-driven GFP is specifically expressed in the peripheral trigeminal and DRG, but not the external cuneate nucleus or the cerebellum (Supplementary Fig. 7). In summary, both *Asic3*^*−/−*^ and *Pv-Cre::Asic3*^*f/f*^ mice showed similar deficits in the balance beam walking task. Moreover, this phenotype could not be recapitulated in the *Nav1.8-Cre::Asic3*^*f/f*^ mice. Together, these immunohistochemical, electrophysiological and behavioural data indicate that in mouse, ASIC3 plays an important role in dynamic aspects of mechanotransduction, likely to be particularly important in fine-tuning proprioception.

## Discussion

In the present study, we took a genetic approach to labelling ASIC3-expressing DRG neurons and their peripheral terminals in mice. ASIC3 is expressed in ∼30% of the total DRG neurons and among these, ∼50% are large, myelinated neurons, ∼30% are small-CGRP-positive peptidergic neurons and ∼20% are small, IB4-positive, nonpeptidergic neurons (Fig. 1). In other words, ASIC3 is expressed in 15% (=30 × 50%) of total DRG neurons that are N52 positive, 9% (=30 × 30%) of DRG neurons that are IB4 positive and 6% (=30 × 20%) of DRG neurons that are CGRP positive. Determining the proportions of ASIC3-expressing neurons is important for interpretation of the phenotypes observed in ASIC3 knockout mice. Dirajlal *et al*. reported that in the wild-type C57BL6 mouse lumbar DRG, ∼47% are N52-positive large diameter neurons and ∼30% are IB4-positive small diameter neurons, whereas ∼23% are IB4-negative, small diameter neurons^37^. We can estimate that ASIC3 is expressed in ∼32% of the large, N52-positive, myelinated DRG neurons, in 30% of the small, IB4-positive neurons, and in 25% of the small, CGRP-positive neurons. This heterogeneity, as well as the stimulus paradigm used, may account for some of the reported differences between the present and previous studies on a possible mechanosensitive role for ASIC3. For example, using soma indentation as the stimulus, Drew *et al*. compared the mechanically activated current between *Asic3*-null and wild-type DRG neurons^12^. They concluded that there is no difference in current amplitude or kinetics between *Asic3* mutants and controls, even when neuronal subpopulations were categorized by cell size, AP duration and IB4 immunoreactivity. One possible explanation for these divergent conclusions is that even in a specific subpopulation (classified by cell size or IB4 expression), the majority of neurons sampled do not express ASIC3.

At least three studies report the expression of ASIC3 in rat DRG and trigeminal neurons using immunohistochemical methods. They suggest that ASIC3 is expressed in large-sized neurons, in either Pv+ trigeminal neurons^25^ or Pv-negative DRG neurons^20^ or in small-sized, mostly peptidergic-nociceptors/metaboreceptors^23^,^38^. Thus, evidence for the expression of ASIC3 in Pv+ neurons is conflicting. The strongest evidence against ASIC3 being expressed in Pv+ neurons comes from patch-clamp studies, indicating that acid-induced currents in masseter afferents from the mesencephalic nucleus of the 5th cranial nerve (MeV) show slow inactivation, which is not typical of ASIC3-like kinetics (rapid desensitization, <500 ms) in skin^15^, muscle^20^ or cardiac^17^ afferents. In our *eGFP-f* knockin mice, we demonstrate clearly that ASIC3 is expressed in most of the DRG Pv+ neurons (including proprioceptors) and proprioceptor terminals of soleus muscle spindles (Fig. 2). Single-cell RT–PCR further supported the expression of ASIC3 mRNA in these Pv+ DRG neurons, in agreement with *in situ* hybridization evidence from rat DRGs^39^. By comparing the acid-induced inward current between wild-type and *Pv-Cre::Asic3*^*f/f*^ Pv+ neurons, we demonstrate clearly here that the fast, acid-induced inward current is mediated, for the most part, by ASIC3. Several possibilities could account for this difference: (i) the lack of ASIC3-immunoreactivity in Pv+ neurons may be due to limited specificity of ASIC3 antibodies, leading to a false-negative result^14^; (ii) the *Accn3* gene can encode different transcript isoforms by alternative splicing in c-terminal exons^40^,^41^,^42^, which could explain inconsistences in ASIC3 antibody labelling between studies; (iii) ASICs are known to exist in heteromultimers in sensory neurons^16^, thus ASIC subtype compositions might differ in DRG proprioceptors. In a recent study in rat, Simon and colleagues demonstrated that spindle afferent discharge is sensitive to the non-selective DEG/ENaC blocker amiloride^35^. Based on this pharmacology, plus immunofluorescence and western blotting results, they proposed α-ENaC, β-ENaC, γ-ENaC and ASIC2a might participate in the stretch-evoked afferent discharge. We show here that ASIC3 is also present and single-cell RT–PCR analysis suggests at least ASIC1b and ASIC2b are also expressed in Pv+ DRG neurons. It is possible, therefore, that heteromultimeric complexes of these channels form the final mechanosensory complex, giving a range of sensitivities and ranges of responses.

The molecular mechanism of mechanotransduction of proprioceptors is largely unknown, at least partly because it has proven so challenging to study. One common approach is to use sophisticated *in vitro* techniques to measure the electrophysiological responses to mechanical stimulation of neurites from dissociated DRG neurons growing in the culture. However, most such *in vitro* studies use a whole-cell mechano-clamp technique to probe the mechanically activated currents, in which stimulation is directly applied to the neuronal soma or neurite cultured on a rigid substrate (for example, glass)^43^. This mechano-clamp approach does not mimic the elastic environment that sensory terminals occupy in muscle or tendon, particularly the precise modality of nerve-terminal stretching^44^. One possible solution is to grow neurite-bearing DRG neurons on an elastic micropillar array with defined spring constant, and stretch neurites by pillar deflection^45^. However, this requires a complicated microfabrication to cast the pillar array and a sophisticated piezo-driven nanomotor to move a single pillus to stretch a neurite. To mitigate these limitations, we took advantage of a well-established *in vitro* model system developed in our laboratory^33^,^46^, to culture neurite-bearing DRG neurons on the fibronectin-coated, elastic PDMS substrate. In this system, we can apply two different mechanical modes to an individual neurite: the SDNS (stretching) mode, where the PDMS substrate is indented with a glass pipette at a fixed distance (15–25 μm) from the distal neurite, or the DNI (pressing) mode, in which the pipette directly indents the neurite. We found that in response to SDNS, genetic ablation or pharmacological inhibition of ASIC3 markedly impeded a Pv+ DRG neuron's ability to generate APs to neurite stretch. In contrast, *Asic3* ablation had no effect on responses evoked in the DNI mode, when a distal neurite is directly touched/pressed. The clear difference in mode dependency of these effects is interesting. Two possible differences between these modes are: (i) the magnitude and modality (ECM-tethered stretching versus nonspecific pressing) of force transmitted to the neurite; and (ii) the involvement of ECM tethering. The way force is delivered to a given neurite in DNI mode is different from the SDNS mode. Our experiments suggest DNI-delivered force might activate other mechanosensitive channels, such as transient receptor potential channels or piezo1/2 (refs 27, 30, 47). In contrast, it is possible that ASIC3, as a mammalian homologue of *Mec-4*, will only activate when the force is delivered indirectly, via ECM tethers, during the deformation of the ECM in SDNS mode. However, the importance of tethers in mammalian systems is unclear, as the mechanosensitivity of piezo1 was affected by cytoskeleton F-actin disruption (with cytochalasin D) in the whole-cell recording mode, but usually unaffected in the patch recording mode when expressed in HEK293 cells^48^. Future studies should examine the subcellular expression of ASIC3 in these Pv+ DRG neurons and test whether manipulation of some key component(s) of ECM or the cytoskeleton can affect the SDNS-induced generation of APs.

In contrast to the striking deficit in touch sensitivity observed in *C. elegans* with mutation of two ASIC3-related DEG/ENaC family genes, *Mec-4* or *Mec-10*, subtle or quite variable mechanosensory phenotypes have been reported in mice lacking particular ASIC subtypes^4^. Previous studies of skin-nerve preparation had shown that the sensitivity of rapidly adapting mechanoreceptors was increased while sensitivity of A-fibre mechanonociceptors was decreased in ASIC3-null mice^6^,^49^. In a colon-pelvic nerve preparation, Jones *et al*. reported mice lacking *Asic3* showed an ∼50% decrease of sensitivity to colon distension compared with wild types^8^. Although these *ex vivo* studies suggest a role for ASIC3 in neural mechanotransduction, *in vitro* mechano-clamp studies testing different subsets of dissociated DRG neurons in culture failed to show any involvement of ASIC3 in mechanically activated current. DRGs from neither *Asic2* nor *Asic3* knockout mice showed significant effects on soma indentation-induced (DNI-like) currents^12^,^13^. This inconsistency between *in vitro* and *ex vivo* studies might be due to the fact that the previous *in vitro* study did not precisely measure mechanotransduction in ASIC3-expressing neurons, in terms of neuron subtypes, mechanical stimulus, ECM tethering and ASIC3 expression^12^,^34^,^50^. Moreover, the whole-cell mechano-clamp approach may only reveal mechanotransduction involving Piezo proteins, and not ASIC3-dependent mechanotransduction^47^,^51^. The present study was designed to address these potentially confounding factors for probing ASIC3-dependent mechanotransduction. First, we genetically labelled the ASIC3-expressing DRG neurons. Second, we adopted an alternative approach to assessing neurite stretch-induced mechanotransduction; that is, by localized elastomeric matrix control^33^,^46^. We found DRG neurons lacking *Asic3* demonstrated a specific form of mechanosensory deficit during the dynamic phase (the response to SDNS: neurite stretching) but not others (that is, DNI: neurite indentation). This mechanosensory deficit of *Asic3* KO during dynamic stretch was also evidenced in *ex vivo* recordings of muscle spindle. The combination of enhanced on-responses (increased firing during stimulus application) with impaired off-responses (fewer APs during release and post-release) may explain some of the apparent discrepancies in the literature. The differences between wild-type and *Asic3* KO proprioceptive responses in isolated muscles were subtle, but consistent (Fig. 6); originally anticipating larger effects, we had earlier carried out a series of experiments with the specific blocker APETx2, using a longitudinal protocol (data not shown). Confounding factors arising from the protocol are always present in such studies, and may have been responsible for the lack of a clear effect of APETx2 at either 500 nM or 1 μM on final analysis. Certainly, however, any effect of APETx2 must have been small, consistent with the subtle effects of *Asic3* KO itself, but further study of this point must await future experiments.

Previous studies in our laboratory have demonstrated that conventional *Asic3* knockout mice displayed normal locomotor activity in a novel open-field chamber, and their rotarod performance is indistinguishable from wild-type control even when the rotating speed is accelerated to 50 r.p.m. (ref. 21). Here we checked in detail whether *Asic3* knockout mice show behavioural impairment in two higher-resolution proprioception tasks: grid- and balance-beam walking. Consistent with our previous study, conventional *Asic3* knockouts performed normally in grid walking and improved normally with practice over three consecutive trials. Strikingly, however, mice with a targeted proprioceptor *Asic3* deletion showed deficits in grid walking in the dark, suggesting they were using visual cues to compensate for an underlying proprioception deficit. Similarly, in the balance-beam task, we only observed a significant genotype difference when the beam width was reduced to 6 mm. Burnes *et al*. reported that *Asic3* knockout mice showed enhanced muscle fatigue, indicated by grip-strength weakness, but only after a prolonged (>3 h) rotarod forced run. Grip strength was normal in rested *Asic3* knockout mice^52^. Thus, the defect we observed seems not to be due to reduced motivation or increased muscle fatigue, as all animals completed the triplicate trials on different beam widths within 3 min. Although central synaptic dysfunction is unlikely to be involved^14^,^21^,^53^, we cannot exclude a contribution from other proprioceptors (GTO, joint and cutaneous afferents). However, it is certainly consistent with deficits detected in the muscle spindles. The spindle involvement is also consistent with observations in diabetic mouse models^54^. These mice, after diabetes induction with streptozotocin, displayed progressive sensorimotor dysfunction and proprioception deficits in the balance-beam task. This phenotypic similarity could be explained if mice without ASIC3 in the present study suffer reduced mechanical sensation of muscle stretch while retaining an intact vestibular or cerebellar motor-coordination system. Among muscle afferents, the *Asic3*-null phenotype must involve low-threshold proprioceptors as it was shown only by *Pv-Cre::Asic3*^*f/f*^ but not by *Nav1.8-Cre::Asic3*^*f/f*^ mice.

Using electrophysiological, immunohistochemical and behavioural data, we provide strong evidence that ASIC3 proteins are a molecular determinant in mediating some aspects of mechanosensation in Pv+ neurons. However, we cannot exclude possibilities such as ASIC3 being required to tether mechanosensitive channels (for example, Piezo2) to ECM^4^,^30^,^49^. The next question is to know whether ASIC3 is a mechanotransducer, that is, a mechanosensitive ion channel or a component of such a channel, in proprioceptors. To determine whether ASIC3 fulfills the criteria of a major primary mechanotransducer, additional evidence is needed. For example, heterologous expression of the candidate channel should result in the acquisition of mechanosensitivity and its biophysical properties should align with those of the endogenous mechanotransducing current^47^. We have demonstrated ASIC3 is involved in generating the neurite stretch-induced mechanotransducing currents in Pv+ DRG neurons. Further studies should try to reproduce the SDNS-induced ASIC3-mediated mechanically activated currents in a heterologous expression system. However, reproducing the mechanosensitivity of ASIC3 in a heterologous expression system might be very challenging, as any proposed mechanotransducer might be a complex of the transducer channels and many accessory proteins, plus specific ECM components^3^,^4^,^55^. Indeed, recent evidence suggests piezo2 plays a major role in such a complex, despite its biophysical properties not aligning with those of the endogenous mechanotransducing current^30^,^56^. The importance of ECM interaction is highlighted by studies showing that stomatin-domain proteins can interact with ASIC channels and modulate nociceptor mechanosensitivity^49^,^57^. To reconstruct the mechanotransducing complex in a heterologous system, a further challenge is that ASIC3 may need to be expressed in a neurite-like structure.

## Methods

### Animals

For Taiwan, all animal procedures followed were in accordance with the Guide for the Use of Laboratory Animals (National Academy Press) and were approved by the Institutional Animal Care and Use Committee of Academia Sinica. In the United Kingdom, animal work was in accordance with the Animals (Scientific Procedures) Act, 1986, Amendment Regulations, 2012, and approved locally at the University of Aberdeen by the Animal Welfare and Ethical Review Board. Detailed information to generate and characterize *Asic3*Δ^*EGFPf*^ and *Asic3*^*f/f*^ mice can be found in the Supplementary Materials. For *Asic3* conditional knockout mice breeding, *Cre*- and *reporter*-transgenic lines were backcrossed to C57BL6 background and kept as heterozygotes for all experiments.

### Generation of *Asic3*-knockout/*eGFP-f*-knockin mice

To generate the targeting vector, an 18.4-kb 129/Svj genomic DNA plasmid clone containing the mouse *Accn3* gene was used as starting material to construct the targeting vector. A 6-kb *Hin*cII∼*Hin*cII DNA fragment, with the 3′-*Hin*cII (H) site located 82 bp downstream the transcription start site (ts, dark blue arrow) and 240 bp upstream the ATG translation start site (light blue arrow) of *Accn3* was used as the 5′- homologous arm. The positive selection cassette pGK-neo was flanked by two Frt sites (light blue oval) and inserted downstream the eGFP-f cDNA (Clontech). These two constructs were then loxP-flanked (red triangles) and followed by the cDNA of wheat germ agglutinin (WGA, a gift from Dr Yoshihara). The 3′-homologous arm is a 4.4-kb *Bam*HI∼*Kpn*I fragment and the *Bam*HI site is located in the 3′-end of exon 2 of mouse *Accn3* gene. ES cell (R1) gene targeting and chimeric male mice were generated in the Transgenic Core Facility of the Academia Sinica.

### Generation of *Asic3* ^*f/f*^ mice

In the targeting vector, two loxP sites (red triangles) flanked Exon1 of *Accn3* (large blue rectangle), which contains the translation initiation codon and one-fourth of the protein-coding sequence. The positive selection cassette *pGK-neo*, flanked by two Frt sites (yellow ovals), was inserted into Intron1 in ES cells. Eight ES cell clones showed homologous recombination of both the 5′-arm and 3′-arm, and two ES cell clones were further identified by genomic DNA PCR to make sure the integration of the loxp site into the 5′-promoter region. These two ES cell clones (#172 and #303) were then used to generate chimeric mice. ES cell (R1) gene targeting and the generation of chimeric male mice was performed in the Transgenic Core Facility of the Academia Sinica.

### Immunostaining

Immunostaining procedures for eGFP-f in the DRG and peripheral muscle were modified from Zylka *et al*.^58^. Briefly, adult mice (12–16weeks old) were anaesthetized with pentobarbital and perfused transcardially with 25 ml of 0.02 M phosphate-buffered saline (pH=7.4, at 4 °C) and then 25 ml of cold fixative (4% (w/v) formaldehyde, 14% (v/v) saturated picric acid, 0.1 M PB (pH=7.4) at 4 °C). Lumbar spinal cord, gastrocnemius muscle and soleus muscle were dissected and post-fixed for 1 h in the same fixative at 4 °C; DRGs were post-fixed for 10 min at 4 °C. Tissues were cryoprotected in 20% sucrose in 0.1 M PB (pH=7.3) at 4 °C for 24 h. After embedding in optimal cutting temperature (OCT) compound, tissues were frozen on dry ice and sectioned immediately in a Leica CM3050S at 12–16 μm. Slides were stored at −80 °C for later usage. For immunostaining, slides were first dried at room temperature for 10 min and then fixed in 4% paraformaldehyde in 0.02 M PBS for 10 min. Sections were washed three times with PBST (PBS+0.1% Triton X-100) and blocked for 1 h in PBST containing 3% bovine serum albumin and 5% normal serum of the secondary antibody's host. Primary antibodies were diluted in blocking solution and incubated overnight at 4 °C. Sections were then washed three times with PBST and incubated for 1 h at room temperature with secondary antibodies (1:250). For mouse GTO, P7 mouse embryos were collected and anaesthetized with 13% urethane (i.p.). The lower trunk was dissected and post-fixed for 2 h in ice-cold 4% paraformaldehyde (PFA), cryoprotected in 30% sucrose o/n and embedded in OCT. Cryosections at 100 μm thick were collected in 12-well plates with removable baskets. After a PBS wash and blocking, trunk sections were incubated with the first antibody for 72 h and second antibody for 24 h at 4 °C. For DAB-Nickel anti-GFP staining, PFA-fixed mouse brain was sectioned with a vibratome at 100 μm thick, and incubated with rabbit anti-GFP antibody (1:5,000) o/n at 4 °C. After three PBST washes, sections were incubated with biotinylated goat-anti-rabbit 2rd antibody (1:500) and then Avidin-Biotin complex (Vector Labs). Positive immunoreactivity signals were visualized using a Nickel-DAB method. Antibodies and titres used in this study are rabbit-anti-GFP (1:1,000, Abcam); chicken-anti-GFP (1:1,000, Aves Lab); mouse-anti-N52 (1:3,000, Sigma); rabbit-anti-peripherin (1:3,000, Chemicon); Biotinylated-IB4 (stock: 400 μg ml^−1^, 1:1,000, Invitrogen); goat-anti-CGRP (1:1,000, Serotec); rabbit-anti-PGP9.5 (1:1,000, Ultraclone); goat-anti-parvalbumin (1:1,000, Swant); rabbit-anti-NF-H (1:2,000, Chemicon).

### Culture of DRG neurons

Mice at the age of 12–16 weeks were killed with CO_2_ and their DRGs were collected and cultured as previously described^22^. Briefly, the dissected DRGs were digested with 0.125% collagenase (type I, Sigma) for 1.5 h at 37 °C and then trypsin for 20 min at 37 °C in 1 × HBSS. The dissociated neurons were triturated via a flame polished Pasteur pipette and seeded on poly-L-lysine-coated cover slides or fibronectin-coated PDMS. DRG neurons were cultured in 3.5 cm Petri dish with DMEM plus 10% fetal calf serum and were maintained in a 5% CO_2_ incubator at 37 °C for 2 days. The cultured DRG neurons were then subjected to electrophysiological recording of acid-induced currents or indentation-induced APs.

### Single-cell RT–PCR

Cultured DRG neurons were identified by the presence of a GFP signal with a microscope in the recording chamber. We used a recording pipette (for patch clamp) to hold a GFP-positive neuron and drew it away from the poly-L-lysine-coated coverslip towards the surface of external solution. Then another capillary pipette (0.86 mm inner diameter) filled with external solution was used to collect the cell. The cell was directly stuck onto the inner wall of the collecting capillary. After collection, the cell was put in a PCR tube containing 10 μl collection solution (0.4 μl of 100 μM oligo-dT, 0.4 μl of 100 μM random hexamer, 1μl 10 mM dNTP, 0.2 μl of 100 mM dithiothreitol, 0.5 μl of RNase inhibitor (40 U μl^−1^) and 7.5 μl of distilled water) and immediately stored at −80 °C for further reversed transcription (RT). For RT reaction, the sample was denatured at 65 °C for 5 min, put on ice for 5 min and mixed with 10 μl RT solution (4 μl of 5 × RT buffer, 0.8 μl of 100 mM dithiothreitol, 0.5 μl of RNase inhibitor, 0.5 μl of SuperScript III (Invitrogen) and 4.2 μl of distilled water). After incubation at 50 °C for 60 min, the RT reaction was stopped by heating at 70 °C for 15 min. The resulting cDNA products were used for PCR reaction. To evaluate the quality of the RT reaction, we used 1 μl of the cDNA product to check the expression of the housekeeping gene GAPDH in 40 cycles of PCR amplification. Samples with signal of GAPDH were selected and processed for two-step, nested PCR with intron spanning primers for ASIC1a, ASIC1b, ASIC2a, ASIC2b, ASIC3, Piezo1, Piezo2 and parvalbumin (for detail of the sequences, see Supplementary Table 1).

### Electrophysiology

*Whole-cell patch-clamp recordings*. The acid-induced inward currents in the GFP-positive DRG neurons were measured as previously described^22^. The perfusion fast-steper (Warner, SF-77B) was attached onto the six-channel valve (Warner, VC-6) to apply acidic artifical cerebrospinal fluid (ACSF) (with or without amiloride). To probe neurite mechanotransduction, we cultured neurite-bearing DRG proprioceptors on a fibronectin-coated PDMS substrate as previously described^33^,^46^. Briefly, we prepared the PDMS substrate on 12-mm coverslip with a 35:1 ratio of base to curing agent. Before seeding the DRG neurons, the PDMS-covered coverslips were exposed to ultraviolet light for 15 min and then coated with fibronectin (Sigma-Aldrich, 10 μg ml^−1^, 150 μl) for 2 h. Whole-cell patch-clamp recordings of mechanotransduction were conducted on neurite-bearing DRG neurons cultured for 48–72 h. The recording pipette (with a resistance of 2–4 MΩ) was filled with internal solution (100 mM KCl, 2 mM Na-ATP, 3 mM Na-GTP, 10 mM EGTA, 5 mM MgCl_2_, and 40 mM HEPES, adjusted the pH to 7.4 with KOH; osmolality 300–310 mOsm) and the neurons were bathed in external ACSF solution (130 mM NaCl, 5 mM KCl, 1 mM MgCl_2_, 2 mM CaCl_2_, 10 mM glucose and 20 mM HEPES, pH=7.4). Mechanical stimuli were delivered by a mechanical probe on an electronically controlled micromanipulator (Scientifica, Patchstar pc8200c). The mechanical probe was microforge polished (Narishige, MF-900) to a resistance of 4–5 MΩ. After loading with ACSF, the mechanical probe was held at −20 mV by a Multiclamp 700B (Axon instrument) in order to monitor the contact with PDMS surface. Once the mechanical probe was lowered down and touched the PDMS surface, a current drop was observed indicating the increased resistance. Thus, we could synchronize the timing of whole-cell recording with the starting point of PDMS indentation. For a stretch-induced AP recording, the mechanical probe was positioned around the distal neurite terminal at an area on the PDMS surface that fulfilled the following criteria: (i) the area was devoid of any other cell (for example, glia or fibroblasts); (ii) the area was at least 100 μm away from the GFP-selected neuron soma; (iii) the area was 15–25 μm away from the distal terminal. To indent the PDMS substrate, the mechanical probe was first advanced to just touch the PDMS surface as an initial position (p0 in Fig. 4d) and then elevated in the *z*-axis for 50 μm (p+50). The indentation of the mechanical probe was programmed to undertake the following steps: (i) descend in the *z*-axis for 150 μm (that is, 100 μm below the initial surface plane, (p-100)) at a velocity of 1 μm ms^−1^; (ii) remain at (p-100) for 400 ms; (iii) elevate at the same velocity to (p+50). For DNI, we used a manual joystick to control the indentation. All of the indentation procedures were monitored by Power1401 MK2 (CED Instrument). A successful recording fulfill the following criteria: (i) the resting membrane potential of a target neuron was initially more negative than −40 mV; (ii) the changes of series resistance was less than 10% throughout the recording; (iii) changes in resting membrane potential were not over 10%; (iv) the overshoot of AP was more than 0 mV.

*Mechanically activated current recording*. For a stretch-induced current recording, the neurons were bathed in external ACSF solution containing tetrodotoxin (300 nM) to eliminate the voltage-gated sodium channel activity. Whole-cell patch-clamp recording was conducted in voltage-clamp mode and with the same mechanical stimulation protocol as in the stretch-induced AP recording, except the indentation velocity was 1.6 μm ms^−1^. To analyse the force dependency of stretch-induced current, a series of indentation depth (25, 50, 75 and 100 μm) were tested within one Pv+ DRG neuron.

*Rheobase analysis of AP threshold*. Although whole-cell patch was formed, the membrane potential was held at −70 mV by injecting current in current clamp mode, a series of stimulus (from −100 pA to 1,000 pA, 100 pA increased for each 12 steps, in a 500 ms duration) was then applied to evoke AP. The minimal current to evoke AP was determined as rheobase current.

*Ex vivo muscle-nerve recording*. The mouse was killed by cervical dislocation. The leg was immediately skinned and the soleus exposed by parting the overlying musculature between the peroneal muscles and gastrocnemius. The leg was then removed at the hip and transferred to a dissection dish and submerged in carbogen (95% O_2_/5% CO_2_)-saturated Liley's saline^59^ at room temperature, which was changed every 15–20 min. The muscle and full length of the nerve up to and including the sciatic were exposed and the soleus muscle length (maximum distance from proximal to distal tendon) measured when the ankle angle was set to 90° relative to the tibia (resting length). The muscle, its nerve back to its exit from the sciatic, and its tendons with short lengths of boney insertion/origin were excised and placed in a PDMS-lined recording chamber. The tibial origin was firmly immobilized on the base and the Achilles tendon insertion was firmly tied to a metal hook on a computer-controlled electromagnetic puller and set to the previously measured *in situ* rest length (generally 9–11 mm). The muscle nerve was taken into a suction electrode and the preparation left to equilibrate for 20–30 min.

A series of 1 mm stretches was then imposed according to the following protocol:

From resting length (*t*=0 min):

Ramp 1=1 s delay, ramp-on (1 s @ 1 mm s^−1^), hold (3 s), ramp-off (1 s @ 1 mm s^−1^), rest (1 s) repeated for 4 cycles. Total, 4 trapezoidal ramps.

0.2 Hz=ramp-on (2.5 s @ 0.4 mm s^−1^), ramp-off (2.5 s @ 0.4 mm s^−1^) × 5, rest (100 ms) repeated for 5 cycles. Total, 25 sawtooth ramps.

1 Hz=ramp-on (0.5 s @ 2 mm s^−1^), ramp-off (0.5 s @ 2 mm s^−1^), × 5, rest (100 ms) repeated for 10 cycles. Total, 50 sawtooth ramps.

5 Hz=ramp-on (0.1 s @ 10 mm s^−1^), ramp-off (0.1 s @ 10 mm s^−1^), × 5, rest (100 ms) repeated for 10 cycles. Total, 50 sawtooth ramps.

Ramp 2=as for Ramp 1.

Total cycle time at rest length ∼6.5 min

Then, ‘optimal' length was set by yernier scale on the electromagnetic puller (resolution 0.1 mm) using the following criteria: (i) the smallest increase in length immediately evoked firing, (ii) firing fell back to baseline from a 1-mm stretch only on reaching the start length again, (iii) any further reduction in length produced no decrease in firing and (iv) these criteria were repeatable for three consecutive 1 mm sawtooth stretches. The firing in response to imposing the pattern of stretches from Ramp 1 to Ramp 2 was then recorded at the new ‘optimal' length.

### Grid-walking task

An elevated 50 × 50 cm^2^ wire mesh with grid size 2 × 2 cm^2^ was used to assess potential proprioception defects^60^. In the bright (house) light condition (260 Lux on the grid measured with digital light meter, model: YF-172, YFE, Taiwan), the mouse was placed in the centre of the mesh and videotaped with a camera below the mesh for 5 min while walking freely on the grid, repeated for three consecutive days. Foot faults were counted when a hindpaw of the tested mouse missed the grid while walking. In the dark condition, the room light was turned off and a dim red bulb (5 W) was projected on the ceiling above the grid. Under this condition (<0.1 Lux on the grid measured with light meter), we videotaped the behaviour of the mice with an infrared-aided camera (X-Loupe UCRL: Ultra Close-up Ring Light, Lumos Technology, Taipei, Taiwan). All data were extracted by an experimenter without previous knowledge of the mouse genotypes in the study.

### Balance beam walking task

The apparatus for beam walking consisted of an uncovered starting stage (white, 15 × 10 cm^2^), a goal box (black, 20 × 20 × 20 cm^3^) and a 1-m beam with a flat surface of 24, 12 or 6 mm width. The beam was set 50 cm above the floor and the time for each mouse to cross the 1-m beam was measured. Male mice between the ages of 12 and 16 weeks were used and housed as groups of five in the test room several days before the training. On the training day, nesting material from the home cage was placed in the goal box to attract the mouse to run across the beam. During training, the mouse was placed on the starting stage and a light was turned on as aversive stimulus to encourage the mouse to cross the beam to the dark goal box. Each mouse learned to cross sequentially the 24 mm beam three times, the 12 mm beam three times and then the 6 mm beam three times with an inter-trial interval of 5 min. Sometimes in the 6-mm trial, the mouse stopped, stalled and looked around on the starting stage without proceeding forward. In these cases, the experimenter encouraged continued forward movement by gently pushing on its tail with a gloved finger. The apparatus was cleaned and wiped with towels soaked with 70% ethanol between trials. The formal test was videoed 48 h after completion of training. Each mouse crossed each width of beam three times in the order: 24, 12 and then 6 mm. Time to transit and the number of hindpaw slips for each width of beam were quantified as index of beam walking performance.

### Statistical analysis

Data in all figures were presented as mean±s.e.m. ‘N' represents the number of mice, whereas ‘n' represents the number of neurons or soleus muscles sampled in a given study. Statistical comparison was performed either by EXCEL (*χ*^2^ analysis) or by SigmaState3.5 (ANOVA). A non-parametric Mann–Whitney test was used for some electrophysiology studies when the raw data did not pass the normal distribution or equal variance test. For all the *post-hoc* tests in ANOVA analysis, the Holm–Sidak method was used, which is more powerful than the Tukey and Bonferroni test. The criterion for a significant difference was set at *P*<0.05.

## Additional information

**How to cite this article:** Lin, S.-H. *et al*. Evidence for the involvement of ASIC3 in sensory mechanotransduction in proprioceptors. *Nat. Commun.* 7:11460 doi: 10.1038/ncomms11460 (2016).

## Supplementary Material

Supplementary Information Supplementary Figures 1-7 and Supplementary Table 1

Supplementary Movie 1 A GFP-positive, Pv+ DRG neurite received stretch via indentation of the PDMS substrate (SDNS) with a blunt pipette at 15 µm away from the distal neurite. The substrate indentation did not generate any discernible movement of the soma during the whole-cell patch clamp recording.

Supplementary Movie 2 Video demonstrating the behavior of a wildtype mouse performing the balance beam task with a beam width of 6 mm.

Supplementary Movie 3 Video demonstrating the ASIC3-deficient phenotype. A Pv+ neuron-specific Asic3 conditional knockout mouse performs the 6mm-width balance beam task. This Asic3 targeted knockout mouse used a very different strategy to its wildtype counterpart (hind leg crawling) to complete the 1-meter long beam walk.

## Figures and Tables

**Table 1:** Results of the statistical analysis of repeated triangular (‘sawtooth') stretches applied to *ex vivo* soleus muscle preparation of wild-type and *Asic3* ^*−/−*^ mice at 3 different frequencies.

**Stretch frequency (Hz)**		**Resting length**			**Optimal length**		
		**F**		***P*-value**	**F**		***P*-value**
0.2	ON	(1,36)=	0.11	NS	(1,36)=	408.81	<0.001
	OFF	(1,36)=	66.81	<0.001	(1,36)=	51.33	<0.001
1	ON	(1,36)=	38.19	<0.001	(1,36)=	274.90	<0.001
	OFF	(1,36)=	57.68	<0.001	(1,36)=	31.00	<0.001
5	ON	(1,36)=	194.59	<0.001	(1,36)=	7.48	<0.001
	OFF	(1,34)=	30.72	<0.01	(1,34)=	1.08	NS

ANOVA, analysis of variance; NS, not significant.

Two-way ANOVA with replicates; values of F and *P* for 1 d.f.
