# Supplementary material for: Evidence for the involvement of ASIC3 in sensory mechanotransduction in proprioceptors
Source: Nat Commun. 2016 May 10;7:11460. doi: 10.1038/ncomms11460 (PMC4866049; doi:10.1038/ncomms11460)
Supplement: Supplementary Information — Supplementary Figures 1-7 and Supplementary Table 1 [file ncomms11460-s1.pdf]

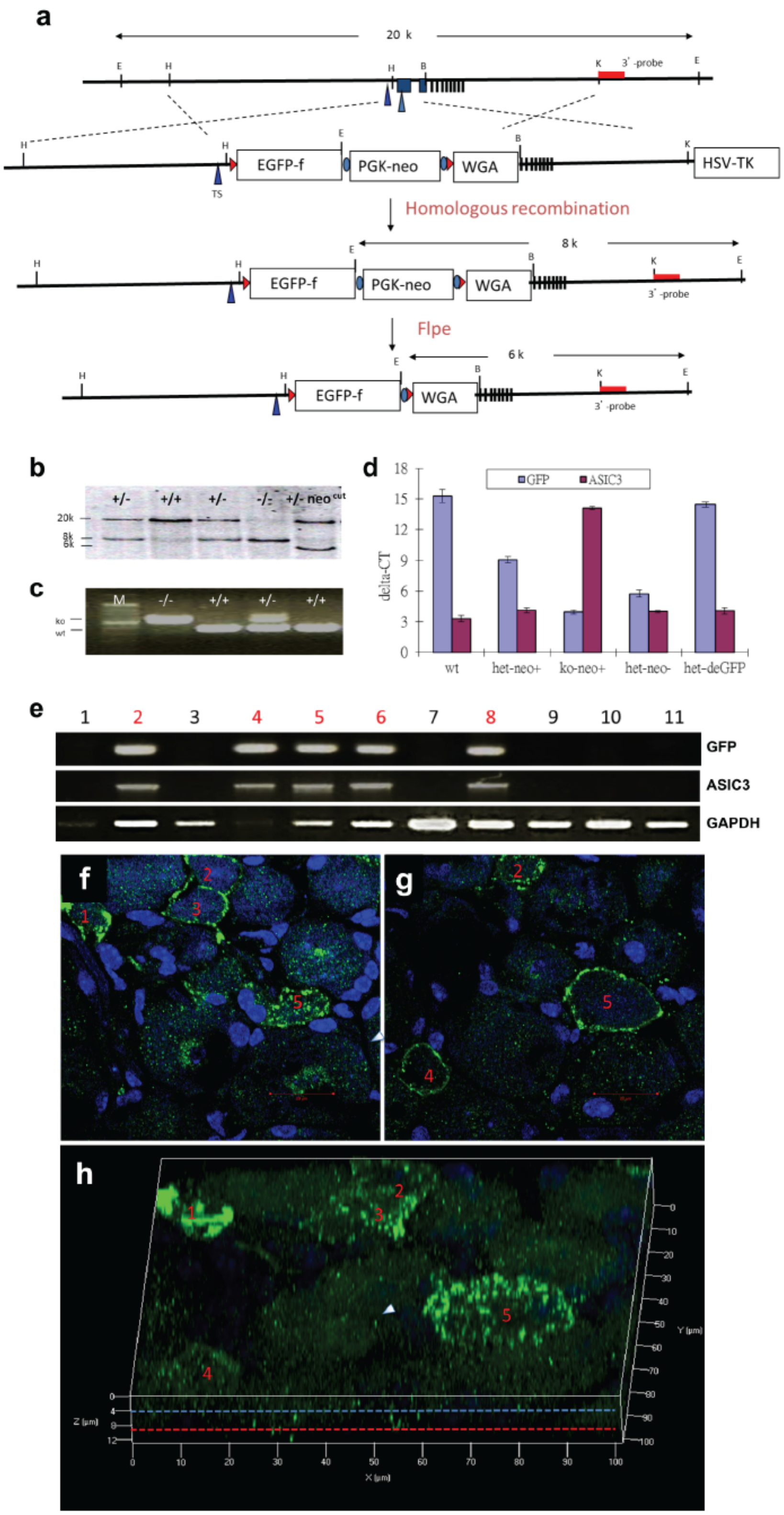

Supplementary Figure 1. Generation and characterization of *Asic3*-knockout /*eGFP-f*-knockin mice. (a) Schematic diagram illustrating the key steps to generate this

mouse. (b) After germline transmission, the pGK-neo cassette was then removed by Flpe-mediated recombination by crossing with Actin-Flpe transgenic mice (Jackson Laboratory, Stock Number: 005703), with the resulting gene deletion verified by Southern blot analysis. (c) Thereafter, mouse genotyping was performed by genomic DNA PCR with the following oligonucleotides: primer 1 (5'-GGGTTTCCTTCAAGCCCTAC-3') corresponding to the 5'-UTR of *Accn3*; primer 2 (5'-GGAGCCAAGACAGGTGAAAG-3') corresponding to the exon1 of *Accn3*; and primer 3 (5'-GAACTTCAGGGTCAGCTTGC-3') corresponding to the open reading frame of *eGFP-f*. The estimated size of the wildtype and knockout alleles was 366bp and 462bp, respectively. Heterozygous progeny were then backcrossed to C57BL/6 for at least 10 generations. (d) To verify the mRNA expression of these genetic targeted genes, we performed real-time Q-PCR to quantify mouse *Asic3* and *eGFP-f* transcripts (for primer sequence, see 'inner' primer sets in Supplementary Table 1). Trigeminal ganglia were dissected and processed for RNA extraction from wildtype (wt), heterozygous with neo cassette (het-neo+), homozygous with neo cassette (ko-neo+), heterozygous without neo cassette (het-neo-) and heterozygous with *eGFP-f* excised mice (het-deGFP) (N=3 for each genotype). The floxed *eGFP-f* was removed by crossing mice with *protamine-Cre* transgenic mice (Jackson Laboratory, Stock Number: 003328). Male offspring bearing the double transgene were then backcrossed with female C57BL/6 to generate *eGFP-f*-null mice. Quantitative real time RT-PCR analyses were followed with the comparative C_T method. Δ C_T was first derived from C_T of target gene- C_T of GAPDH for a given sample. For the expression of ASIC3 transcript, we used Δ C_T of *Asic3* from wild type (*Asic3*$^{+/+}$) sample as baseline and calculated the $2^{-\Delta\Delta\ CT}$ value for each genotype [$\Delta\Delta C_T = \Delta C_T$ (specific knockout)-ΔC_T (*Asic3*$^{+/+}$)]. For eGFP-f transcript, we used Δ C_T of *eGFP-f* from *Asic3*$\Delta^{EGFPf/EGFPf}$ sample as baseline and calculated the $2^{-\Delta\Delta\ CT}$ value for each genotype [$\Delta\Delta C_T = \Delta C_T$ (specific knockout)-ΔC_T (*Asic3*$\Delta^{EGFPf/EGFPf}$)]. Results indicated that the expression of ASIC3 transcript in trigeminal ganglia was less in *Asic3*$\Delta^{EGFPf/+}$ than *Asic3*$^{+/+}$ and was barely detectable in *Asic3*$\Delta^{EGFPf/EGFPf}$. In contrast, the expression of eGFPf transcript was significantly increased in trigeminal ganglia of *Asic3*$\Delta^{EGFPf/+}$ and *Asic3*$\Delta^{EGFPf/EGFPf}$ as compared with *Asic3*$^{+/+}$. (e) To verify whether the expression of *eGFP-f* and *Asic3* are correlated at a single cell level, we cultured heterozygous (neo-) DRG neurons and randomly sampled 16 neurons with a diameter larger than 30μm for single cell RT-PCR. The integrity of the single cell cDNA was first determined by screening for mouse GAPDH with a 35-cycle PCR. Of the 16 single neurons harvested, 11 showed at least some *GAPDH* expression and, as shown here, subsequently underwent two rounds of 35-cycle nested-PCR with the outer and inner primer set specific for mouse *Asic3* and *eGFP-f* (for primer sequences, see Supplementary Table 1). 5 neurons (#2,4,5,6 & 8) were ASIC3-positive and a perfect correlation was observed since eGFP-f was detected only in these 5 ASIC3-positive neurons. (f-h) Immunostaining of eGFP-f in the *Asic3*$\Delta^{EGFPf/EGFPf}$ DRG is demonstrated in the confocal z-axis scans. (f) A representative section shows 4 eGFP-positive cells at a z-position. (g) The same section at different z-position shows 3 eGFP-positive cells and 2 of them (#2 and #5) are identical cells as shown in (f). (h) The 3-D relationships of 5 membrane-tagged eGFP positive neurons shown in (f) and (g). The blue and red dashed-lines in image (h) indicate the z-position of image (f) and image (g), respectively.

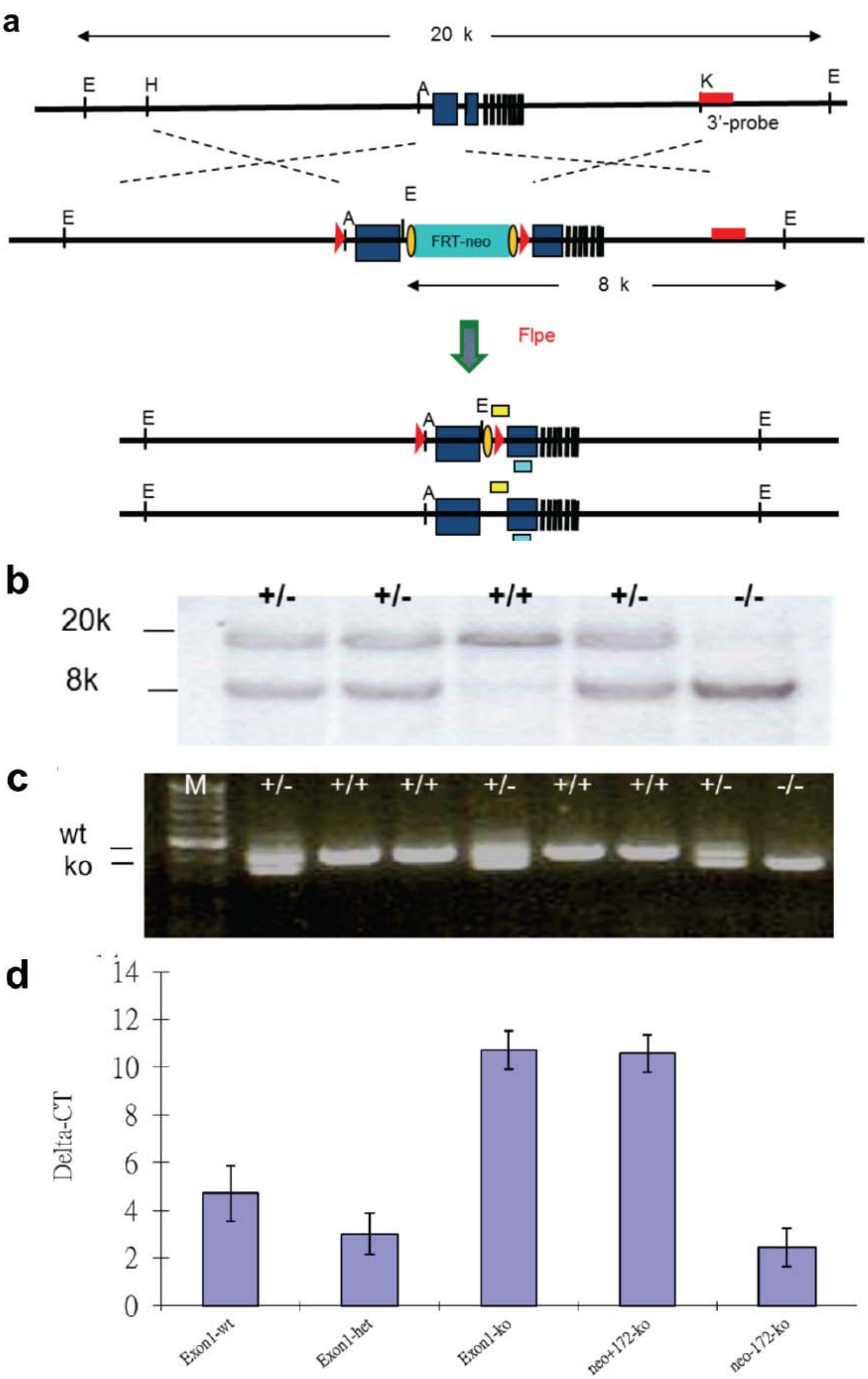

Supplementary Figure 2. Generation and characterization of *Asic3*$^{f/f}$ mice. (a) Schematic diagram illustrating the key steps to generating a floxed allele of mouse *Accn3*. (b) A Southern blot of clone #172 F1 progeny proved the homologous recombination in the mouse *Accn3* locus. Heterozygous progeny were then crossed with the *Actin-Flpe* transgenic mice to excise the selection cassette *pGK-neo*. After

mating the wildtype C57BL/6 for one generation, progeny negative for *neo* and *Flpe* were backcrossed with C57BL/6 mice and genotyped by PCR. (c) Two oligonucleotides: Primer-1 (5'-GATTTGTCACTGCCATGGTG-3') corresponding to the 3'-end of *Accn3* intron 1 and primer-2 (5'-AGCAGGAGGAGTATCTGCC-3') corresponding to the 5'-end of *Accn3* exon 2 were used to generate a wildtype allele (400bp) and the conditional knockout allele (450bp due to the insertion of one additional loxP site). (d) In order to confirm that the 5'-insertion of loxP sequence did not alter normal *Accn3* transcription and the floxed *Accn3* exon1 is excisable upon Cre recombination, we took cDNA from the trigeminal ganglia of floxed and knockout mice to perform real time RT-PCR. The expression levels of ASIC3 transcripts were quantified and compared by using the $2^{-\Delta\Delta\ CT}$ method. We used ΔC_T of ASIC3 from *Asic3*$^{+/+}$ sample as baseline and calculated the $2^{-\Delta\Delta\ CT}$ value for each genotype [$\Delta\Delta C_T = \Delta C_T$ (specific knockout)-ΔC_T (*Asic3*$^{+/+}$)]. Results indicated that before *pGK-neo* excision, the expression of ASIC3 was hampered and decreased to the knockout level. After *pGK-neo* excision, ASIC3 transcripts were equivalent to the wildtype (*Asic3*$^{+/+}$) level, which indicated that the insertion of the 5'-loxP in the promoter region of ASIC3 did not affect normal mRNA transcription in *Asic3*$^{f/f}$ mice. We next crossed the *Asic3*$^{f/f}$ mice with the *Protamine-Cre* mice to check whether ASIC3 transcripts can be eliminated by Cre expression in the germ cells. The results indicated that ASIC3 mRNAs were depleted in *Asic3*$^{-/-}$ mice (offspring of *Protamine-Cre::Asic3*$^{f/+}$ intercrossed) as expected (each bar represents mean±SEM, N=3 for each genotype). As further controls, two further backcrossings to the wildtype C57BL6 mice were carried out to make sure the *Protamine-Cre* allele was removed and kept the knockout mice for future behavioral studies.

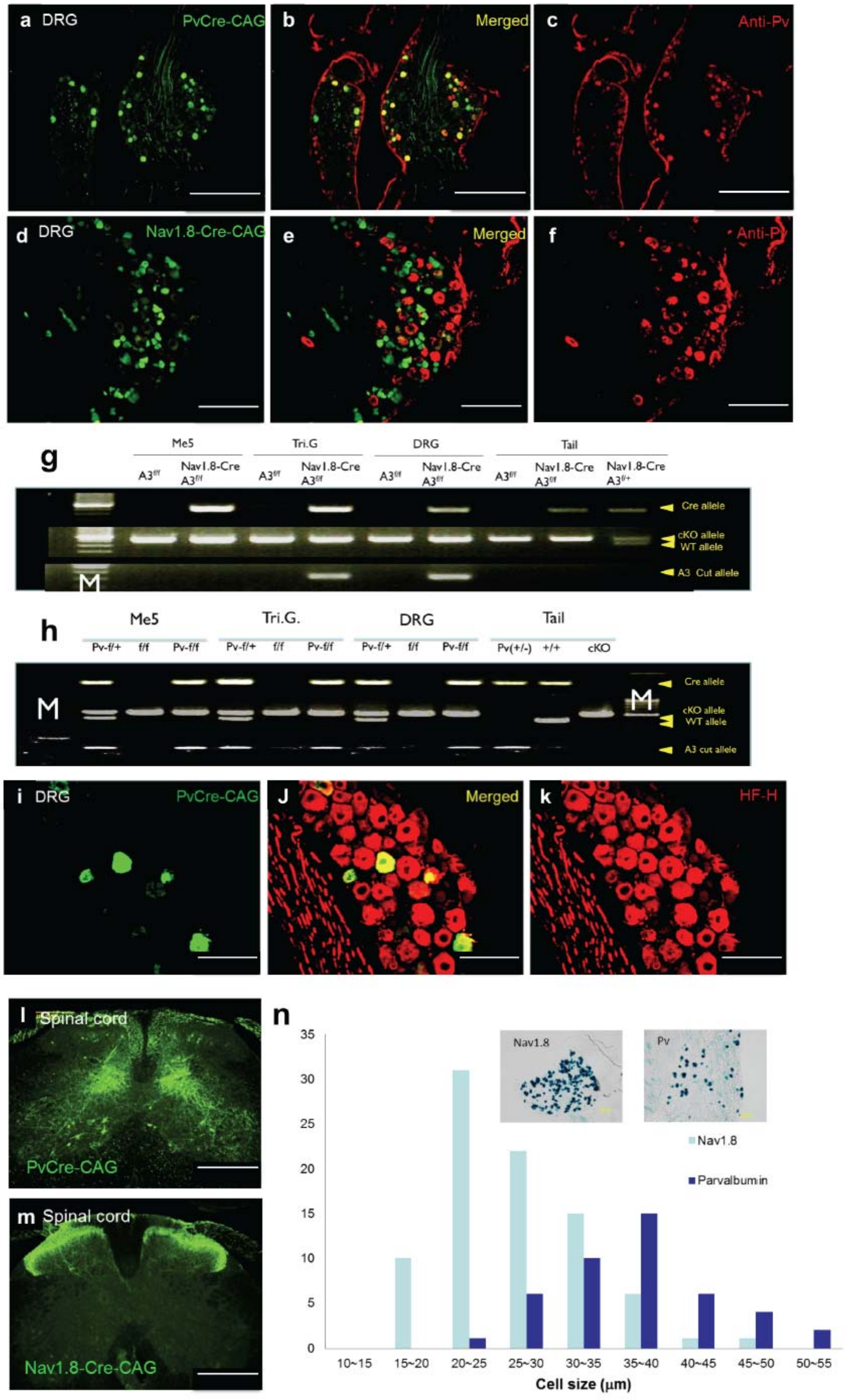

Supplementary Figure 3. Comparison of the expression of Pv+ proprioceptors and Nav1.8-positive nociceptors in lumbar DRG and spinal cord. (a-c) Colocalization of

parvalbumin immunoreactivity and Parvalbumin (Pv)-Cre mediated GFP expression in the lumbar DRG of *Pv-Cre::CAG-STOP*^{floxed}*-GFP* double transgenic mice. 87% GFP-expressing neurons observed expressed parvalbumin. The very high coincidence of Pv immunolabelling and GFP expression validated that the Pv-Cre driven GFP signal used in this study can faithfully represent the expression of Pv (Scale bar = 500 μm). (d-f) In contrast, there is nearly complete and mutually exclusive expression (<0.5% co-expression) of Pv immunoreactivity and *Nav1.8-Cre* mediated GFP expression in the lumbar DRG of *Nav1.8-Cre::CAG-STOP*^{floxed}*-GFP* double transgenic mice (scale bar = 200 μm). (g,h) Cre-mediated *Asic3* conditional knockout in *Nav1.8-Cre::Asic3*$^{f/f}$ and *Pv-Cre::Asic3*$^{f/f}$ mice. We took genomic DNA samples from the mesencephalic nucleus of the 5th cranial nerve (MeV), trigeminal ganglion and the lumbar DRG. PCR of the generic Cre allele was performed according to the genotyping method/primers suggested in the Jackson's laboratory. To verify whether the floxed-*Asic3* allele is excised upon Cre-mediated recombination, we used the forward primer-3 (5'-CTCGAGGCCCACATAACTTCG-3') to target the XhoI-site (bold) and the partial loxp sequence (underline) we generated when introducing the loxP site into the 5'-UTR. Combined the forward primer-3 and the reversed primer-2 (see Supplementary Fig.3c), we could check the *Asic3*/exon1 cut allele (308bp) via PCR. Results confirmed that the *Asic3*-cut allele was appeared in a Cre-dependent manner. (i-k) Comparison of neurofilament heavy chain (NF-H) and *Pv-Cre* mediated GFP expression in the lumbar DRG of *Pv-Cre::CAG-STOP*^{floxed}*-GFP* double transgenic mice (scale bar = 100 μm). Every eGFP-positive neuron (Pv+ DRG proprioceptor) is a large-, myelinated-neuron. These are the type of neurons that have peripheral projections to muscle spindles skeletal muscle (Fig. 2k). (l) Central projections of the DRG proprioceptors visualized by GFP staining in the lumbar spinal cord of *Pv-Cre::CAG-STOP*^{floxed}*-GFP* double transgenic mice. The central projections of these proprioceptors by-pass the superficial dorsal horn to target the premotor interneurons in lamina VI (scale bar = 200 μm). (m) Central projections of the DRG nociceptors visualized by GFP staining in the lumbar spinal cord of *Nav1.8-Cre::CAG-STOP*^{floxed}*-GFP* double transgenic mice. Consistent with previous studies, the majority of central projections from Nav1.8-positive DRG nociceptors target the superficial dorsal horn, typically the inner part of lamina II (scale bar = 200 μm). (n) Cell soma diameter distribution of lumbar DRG proprioceptors and nociceptors. Proprioceptors and nociceptors were counted by x-gal staining of lumbar DRG from Cre mediated β-gal expression in the *Pv-Cre::ROSA-Gt26* and *Nav1.8-Cre::ROSA-Gt26* double transgenic mice, respectively. Lumbar DRGs were fixed, cryoprotected, sectioned at 16μm and processed for X-gal staining (solution composition: 0.15M NaCl, 3.5mM $K_3Fe(CN)_6$, 3.5mM $K_4Fe(CN)_6$, 0.01M PB, pH=7.4, 1mM $MgCl_2$, 0.3M Chloroquine, 0.01% sodium deoxycholate, 0.2% NP-40 and 1mg/ml X-gal) at 37^{0}C for 3hours. The two cell size distributions overlap but indicate that Pv+ proprioceptors are predominantly the large-sized neurons while Nav1.8-positive nociceptors are small-sized neurons.

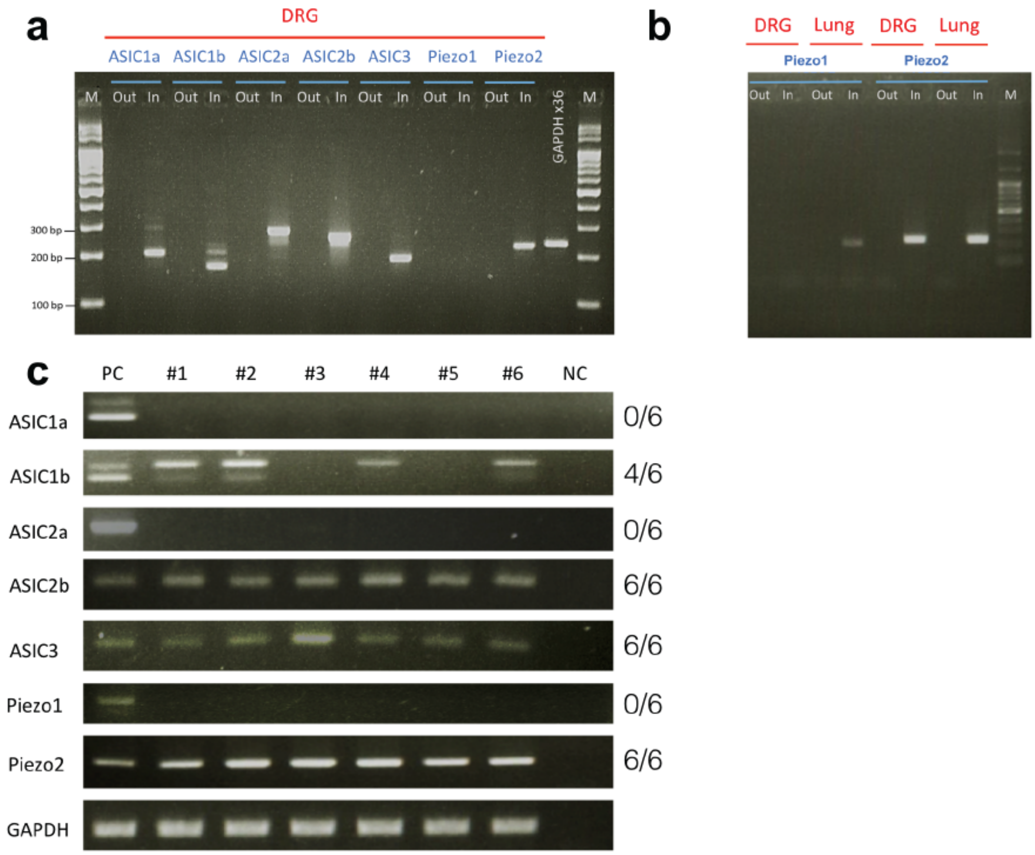

Supplementary Figure 4. Single cell RT-PCR analysis of ASIC and Piezo subtypes in Pv+ DRG neurons. (a) Validation of primer sets for single cell RT-PCR to detect ASIC1a, ASIC1b, ASIC2a, ASIC2b, ASIC3, Piezo1 and Piezo2. L6 lumbar DRG from Wildtype was dissected and extracted for total RNA. 2µg of total RNA were processed for RT reaction. Subsequent RT sample was diluted 1000 folds and processed for two-step nested-PCR to test optimal condition. All targets corresponding to the correct DNA sizes were confirmed after the PCR reaction using inner primers for the five ASIC-subtypes and Piezo2. (b) Due to the fact that Piezo1 was not detectable in the L6 DRG lysate, we validated the Piezo 1 primer set in the RT-product of the lung. Result indicated that nested primers for Piezo1 are useful to detect the expression of Piezo1 in the lung. (c) In 6 single Pv+ DRG neurons, ASIC1b (4/6), ASIC2b (6/6), ASIC3(6/6) and Piezo2(6/6) were detectable. No expression of ASIC1a (0/6), ASIC2a (0/6) and Piezo1(0/6) in the same samples.

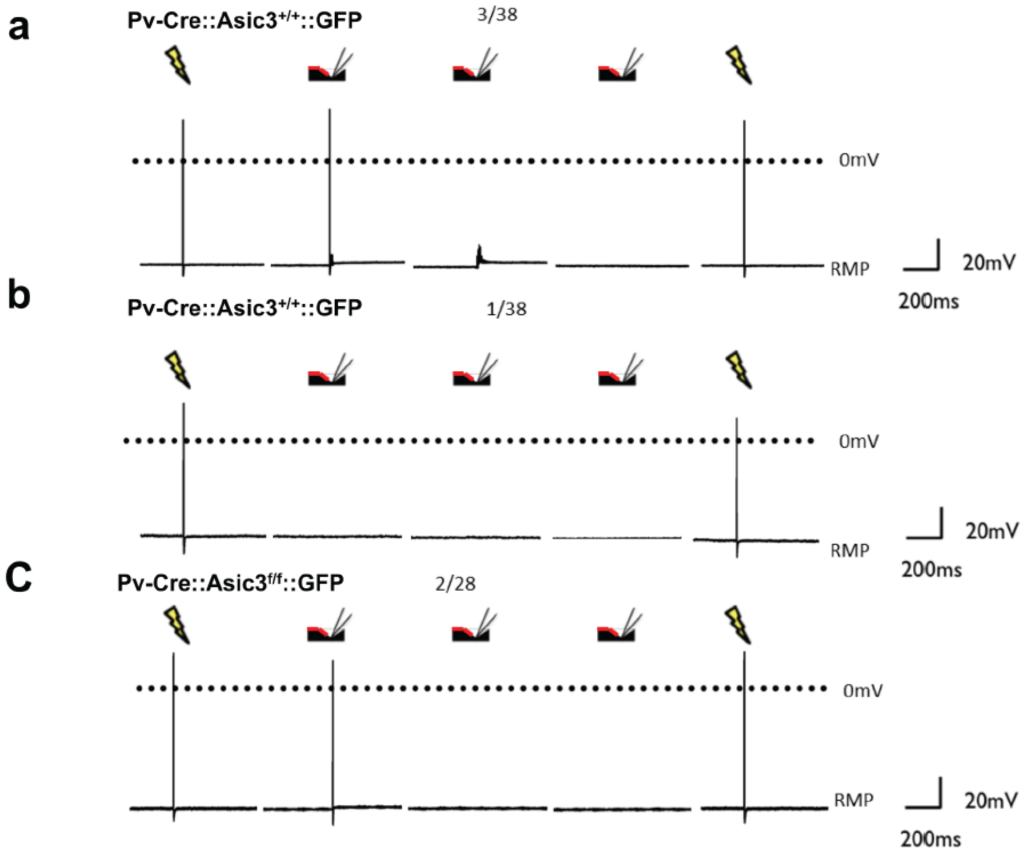

Supplementary Figure 5. Sample recordings from wildtype and *Asic3*-null Pv+ DRG neurons to neurite stretch. (a) In contrast to the wildtype neurons, only 2 of 28 *Asic3*-null Pv+ neurons produced an action potential with substrate-indentation, and then only on the first indentation. In the remaining 26, indentation failed to induce firing in any of the three trials. All neurons analyzed were otherwise healthy, as they produced an AP in response to direct electrical stimulation both before and after the mechanosensory trials (jagged arrow) to be included in the analysis. (b) In 3 wildtype Pv+ neurons, only the first trial produced a neurite-stretch induced action potential, not the subsequent substrate-indentations. Consequently, these 3 Pv+ neurons were classified as non-responders. (c) The remaining wildtype Pv+ neuron produced no SDNS-evoked action potentials in any trial.

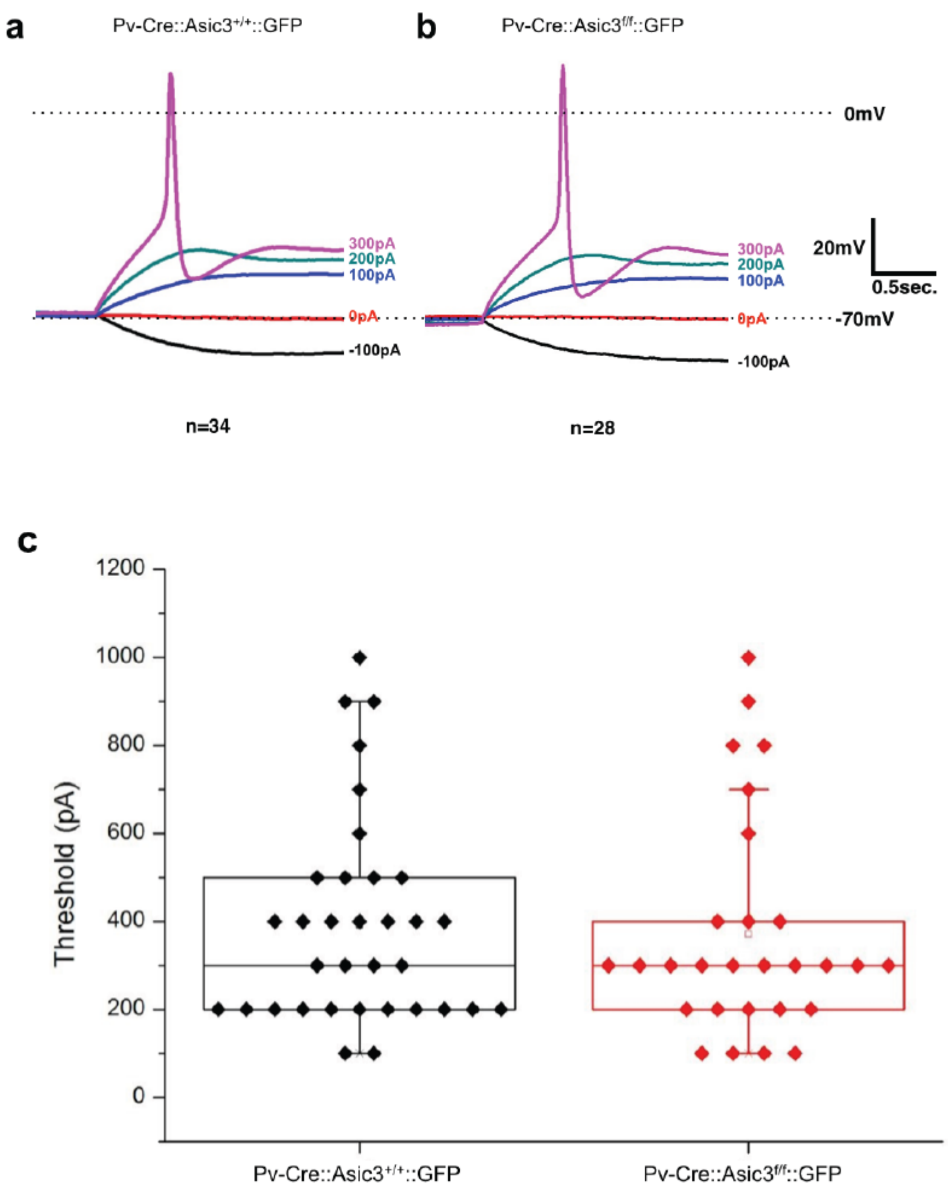

Supplementary Figure 6. Rheobase analysis of action potential threshold in wildtype and *Asic3*-null Pv+ DRG neurons. (a,b) Action potentials were recorded in current-clamp mode with a 100pA step-up current injection protocol. (c) Data analysis comparing neurons from wild type and *Asic3* knockout (*Pv-Cre::Asic3*$^{f/f}$) mice indicated that there was no significant difference in the action potential threshold between genotypes (Mann-Whitney U-test, U=514.5,T=843.5, p=0.584).

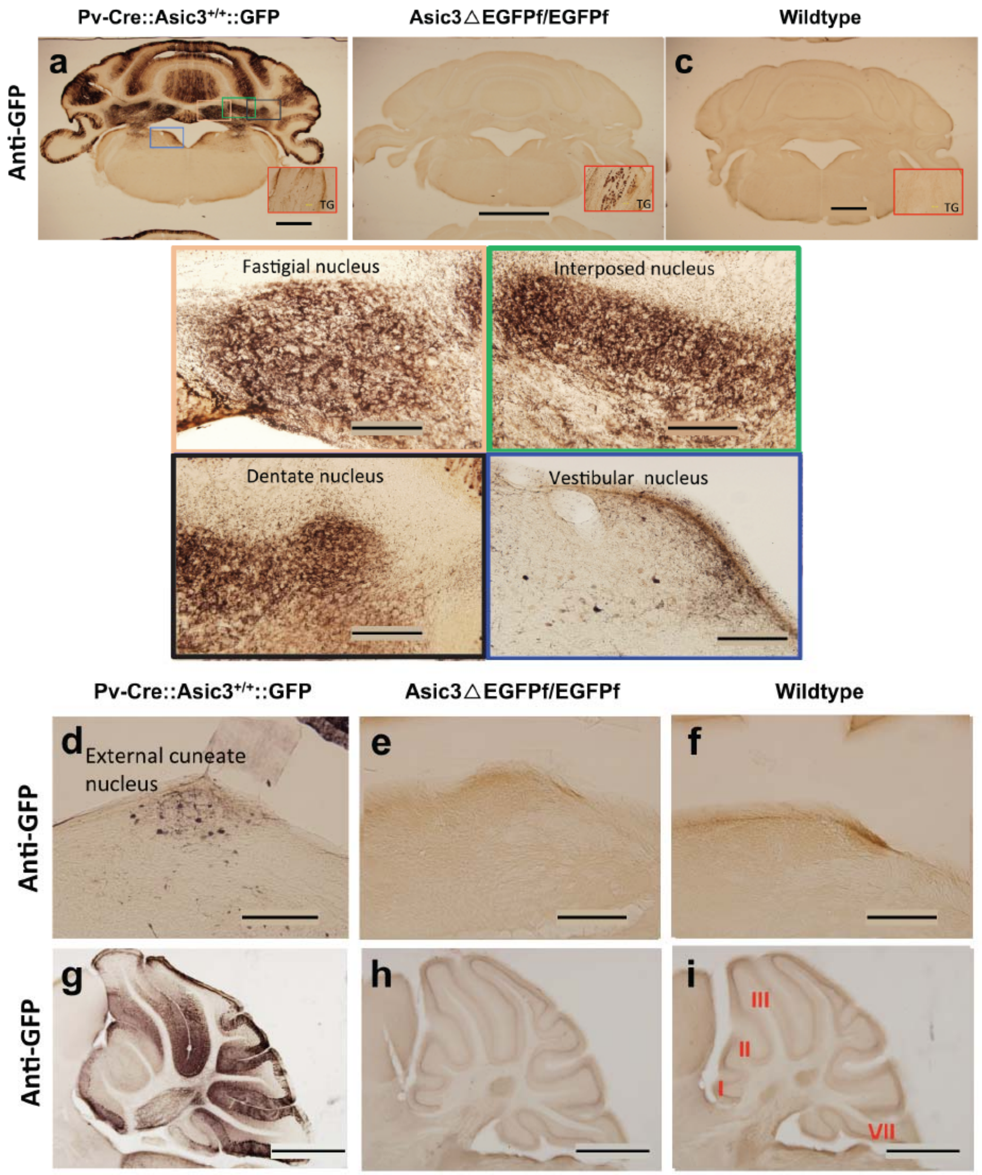

Supplementary Figure 7. Immunostaining for GFP to test for the expression of parvalbumin and ASIC3 in supraspinal proprioception-related nuclei in mice. (a-c) GFP immunoreactivity was observed in Pv-positive cells in the cerebellum of the *Pv-Cre::GFP-reporter* mice (a) and ASIC3-positive neurons in the trigeminal ganglia of the *Asic3*$\Delta^{EGFPf/EGFPf}$ mice (b) but not in wild type mice (c). (d-i) GFP immunoreactivity indicates its expression in Pv-positive cells in several precerebellar nuclei. (j-l) In the external cuneate nucleus, GFP immunoreactivity was observed in Pv-positive cells in the *Pv-Cre::GFP-reporter* mice (i), but not in the *Asic3*$\Delta^{EGFPf/EGFPf}$ mice (k), or in wild type mice (l). Scale bars = 1 mm in (a) & (b), 2 mm in (c), and 200 μm in from (d) to (i).

Supplementary Table 1. Primers of single cell RT-PCR.

**Gene**	**Outer forward**	**Outer reverse**
**Asic1a**	5'-CAGATGGCTGATGAAAAGCA-3'	5'-ATGCTTCTCTCGTGCCACTT-3'
**Asic1b**	5'-CTGTGGTCCCCACAACTTCT-3'	5'-TCGTCCTGACTGTGGATCTG-3'
**Asic2a**	5'-GCCCTGTTGGATGTCAACTT-3'	5'-TCAGACTGGCTGTGGATCTG-3'
**Asic2b**	5'-GCTGCTCTCCTGCAAGTACC-3'	5-'TGAGCCTCTGCTCTTGTGTG-3'
**Asic3**	5'-GTCTGGACCCTGCTGAACAT-3'	5'-GGCAGATACTCCTCCTGCTG-3'
**Piezo1**	5'-CAGATGGCACCCGCCAGAGGTT-3'	5'-TAGCGGAAGACGGCCACCTTCAG-3'
**Piezo2**	5'-CGGTTGCTACAGTCCCTGTGCAT-3'	5'-CAATCAGGTGCCCAGCAGTGAAGA-3'
**Gene**	**Inner forward**	**Inner reverse**
**Asic1a**	5-'CTCCCCACACAGGCAAGTAT-3'	5'-CTCCCCACACAGGCAAGTAT-3'
**Asic1b**	5'-CTGTGGTCCCCACAACTTCT-3'	5'-CTCCCCACACAGGCAAGTAT-3'
**Asic2a**	5'-GTAGGCCATGACCTGAAGGA-3'	5'-TCAGACTGGCTGTGGATCTG-3'
**Asic2b**	5-'GCTGCTCTCCTGCAAGTACC-3'	5'-TCAGACTGGCTGTGGATCTG-3'
**Asic3**	5-'CCCAGTCCGACTTTTGACAT-3'	5'-CCCTTAGGAGTGGTGAGCAG-3'
**Piezo1**	5-'CAGTACCTGCTGTGTTTGGGCA-3'	5'-CGCTGAGAAGACCTGCCACTG-3'
**Piezo2**	5'-GCAAGTCTGACCATCTGGCT-3'	5'-GACAGAGGCGAACCTGCGAA-3'
